# MsrR is a thiol-based oxidation-sensing regulator of the XRE family that modulates *C. glutamicum* oxidative stress resistance

**DOI:** 10.1186/s12934-020-01444-8

**Published:** 2020-10-02

**Authors:** Meiru Si, Can Chen, Jingyi Zhong, Xiaona Li, Yang Liu, Tao Su, Ge Yang

**Affiliations:** 1 College of Life Sciences, Qufu Normal University, Qufu, 273165 Shandong China; 2 College of Life Science and Agronomy, Zhoukou Normal University, Zhoukou, 466001 Henan China

**Keywords:** Oxidative stress, MsrR, Transcription regulation, *Corynebacterium glutamicum*

## Abstract

**Background:**

*Corynebacterium glutamicum* thrives under oxidative stress caused by the inevitably extreme environment during fermentation as it harbors antioxidative stress genes. Antioxidant genes are controlled by pathway-specific sensors that act in response to growth conditions. Although many families of oxidation-sensing regulators in *C. glutamicum* have been well described, members of the xenobiotic-response element (XRE) family, involved in oxidative stress, remain elusive.

**Results:**

In this study, we report a novel redox-sensitive member of the XER family, MsrR (multiple stress resistance regulator). MsrR is encoded as part of the *msrR*-*3-mst* (3-mercaptopyruvate sulfurtransferase) operon; *msrR*-*3-mst* is divergent from multidrug efflux protein *MFS*. MsrR was demonstrated to bind to the intergenic region between *msrR*-*3-mst* and *mfs*. This binding was prevented by an MsrR oxidation-mediated increase in MsrR dimerization. MsrR was shown to use Cys62 oxidation to sense oxidative stress, resulting in its dissociation from the promoter. Elevated expression of *msrR*-*3-mst* and *mfs* was observed under stress. Furthermore, a Δ*msrR* mutant strain displayed significantly enhanced growth, while the growth of strains lacking either *3-mst* or *mfs* was significantly inhibited under stress.

**Conclusion:**

This report is the first to demonstrate the critical role of MsrR-3-MST-MFS in bacterial stress resistance.

## Background

Reactive oxygen species (ROS), including hydrogen peroxide (H_2_O_2_), superoxide anion (O_2_^·−^), hydroxyl radical (·OH), hydroperoxy radical (HO_2_·), singlet oxygen (^1^O_2_), and organic hydroperoxides (OHPs), are inevitable byproducts of aerobic respiration that are also generated under environmental stress by perturbation of the electron transfer chain [1]. ROS can react with the membrane, free fatty acids, and other macromolecules via free radical chain reactions, resulting in the production of a wide spectrum of detrimental carbonyl-containing compounds [2, 3]. The excessive production of ROS is harmful to living systems as it induces oxidative stress and causes subsequent cellular damage to molecules such as DNA, proteins, and lipids [4]. To ensure survival in a hostile environment, versatile resistance defense mechanisms, such as eliminating ROS, deterring the transformation of ROS into more toxic compounds and repairing damaged biomacromolecules, have been developed [5–7]. Low-molecular-weight (LMW) thiols and multiple antioxidant enzymes play crucial roles in defense mechanisms. When bacteria encounter oxidative stress due to a specific ROS, they modulate the expression of the corresponding resistance enzymes [8, 9]. To achieve this, bacteria use pathway-specific transcription factors that act in response to specific ROS and coordinate the appropriate oxidative stress-associated genetic response. Thus, the regulation of antioxidant expression is an important issue. The constant sensing of ROS can be mediated by oxidation of one or more thiolates in regulators [10].

Many of the best characterized bacterial sensors of ROS, such as the LysR (DNA-binding transcriptional dual-lysine regulator) family regulator OxyR (the thiol-based redox sensor for peroxides) [11, 12], zinc-associated extracytoplasmic function (ECF)-type sigma factor H (SigH) [13, 14], the ferric uptake regulator (Fur) family regulator PerR (a peroxide regulon repressor) [15], the MarR (multiple antibiotics resistance regulators) family regulator OhrR (an organic hydroperoxide resistance regulator) [16], the TetR (a tetracycline repressor protein) family regulator NemR (a N-ethylmaleimide regulator) [17], and the AraC (cytosine β-d-arabinofuranoside) family regulator RclR (a regulator of hypochlorous acid (HOCl)-specific resistance) [18], have been shown to contribute to or to modulate antioxidant gene expression [11–18]. These sensors specifically sense ROS via a thiol-based mechanism [11–18]. Upon exposure to oxidative stress, these regulators are activated or inhibited by morphological changes caused by cysteine oxidation, after which they are released from or bind the promoters of target genes, leading to the upregulation of these target genes. Interestingly, more recently, Hu et al. found that the xenobiotic response element (XRE) family transcriptional regulator SrtR (stress response transcriptional regulator) in *Streptococcus suis* is also involved in oxidative stress tolerance, the only report of stress resistance in a member of the XRE family thus far [19]. Unfortunately, its exact molecular mechanism related to oxidant sensing, its target genes, and its interplay with other regulators have not yet been described. XREs, which are widely distributed in living organisms, control the expression of virulence factors, antibiotic synthesis and resistance genes, and stress response genes [20]. Although the XRE family is the second most common family of regulators in bacteria, XRE family members have been reported in only a limited number of bacteria, such as S*taphylococcus aureus* [21], *Rhizobium etli* [22], *S. suis* [19], and *Chloroflexus aurantiacus* [23]. Until now, research on XREs has mainly focused on XREs in eukaryotes. In eukaryotes, the regulatory mechanism of XREs is well known but different from that of ROS-sensing regulators; many xenobiotics acting as inducers, such as oxidants, heavy metals, antibiotics, and toxins, bind aromatic hydrocarbon (Ah) receptors in the cytoplasm to form an Ah receptor-ligand complex, which then interacts with XREs in the nucleus, finally stimulating the transcription of the target genes [24, 25]. However, the functions of XREs in eukaryotes were not reported to be related to oxidative stress or other tolerance to other stresses. Thus, much research about XREs remains to be carried out, especially on the functions and mechanisms of XREs related to oxidative stress and tolerance to other stresses in bacteria.

*Corynebacterium glutamicum*, a nonpathogenic, GC-rich, and gram-positive bacterium, is not only an important industrial strain for the production of amino acids, nucleic acids, organic acids, alcohols, and biopolymers but also a key model organism for the study of the evolution of pathogens [26]. During the fermentation process, *C. glutamicum* inevitably encounters a series of unfavorable conditions [27, 28]. However, *C. glutamicum* thrives under the adverse stresses of the fermentation process using several antioxidant defenses, such as millimolar concentrations of mycothiol (MSH) and antioxidant enzymes [29–32]. Although many thiol-based redox-sensing regulators from different transcription factor families, including LysR (OxyR), MarR [RosR (regulator of oxidative stress response)/OhsR (organic hydroperoxides stress regulator)/CosR (*C. glutamicum* oxidant-sensing regulator)/QorR (quinone oxidoreductase regulator)], TetR [OsrR(Oxidative stress response regulator)], ArsR [CyeR (*Corynebacterium* yellow enzyme regulator)], and SigH, have been well studied [14, 29–31, 33–35], whether the XRE proteins of *C. glutamicum* play a role in protecting against oxidative stress by directly regulating antioxidant genes remains obscure*.* The putative XRE family transcriptional regulator NCgl2679, named MsrR (multiple stress resistance regulator) due to the results of this study, is not only located immediately downstream and in the opposite direction of the multidrug efflux protein NCgl2680 (MFS) but also organized in an operon with 3-Mercaptopyruvate sulfurtransferase (NCgl2678, 3-MST) and the putative protein NCgl2677. This genetic organization allowed us to investigate the function of *C. glutamicum* MsrR in response to environmental stresses. In the present study, MsrR was found to directly control expression of the *msrR*-*3-mst-ncgl2677* operon and the *mfs* gene as a thiol-based redox-sensing transcriptional repressor. The expression of *msrR*, *3-mst* and *mfs* was induced by oxidative stress. MsrR contains only one cysteine residue at position 62 (Cys62). Upon oxidative stress induced by various xenobiotics, MsrR underwent dimerization and lost its DNA-binding activity through the formation of an intermolecular disulfide bond between the Cys62 residue of each subunit. These findings suggest that MsrR is a redox-sensing transcriptional regulator involved in the oxidative stress response of *C. glutamicum* by its regulation of *3*-*mst* and *mfs* expression.

## Methods

### Strains and culture conditions

Bacterial strains and plasmids used this study were listed in Additional file 1: Table S1. *Escherichia coli* and *C. glutamicum* were cultured in Luria–Bertani (LB) broth aerobically or on LB agar plates as previously reported [36]. Δ*msrR*, Δ*3*-*mst* and Δ*mfs* in-frame deletion mutants were produced as described [37]. Briefly, the pK18*mobsacB*-Δ*msrR* plasmid was transformed into *C. glutamicum* wild type (WT) through electroporation to carry out single crossover. The transconjugants were selected on LB agar medium containing 40 µg/ml nalidixic acid and 25 µg/ml kanamycin. Counter-selection for markerless in-frame deletion was performed on LB agar plates with 40 µg/ml nalidixic acid and 20% sucrose [37]. Strains growing on this plate were tested for kanamycin sensitivity (KAN^S^) by parallel picking on 40 µg/ml nalidixic acid-containing LB plate supplemented with either 25 µg/ml kanamycin or 20% sucrose. Sucrose-resistant and kanamycin-sensitive strains were tested for deletion by PCR using the DMsrR-F1/DMsrR-R2 primer pair (Additional file 1: Table S2) and confirmed by DNA sequencing. The Δ*3*-*mst* and Δ*mfs* in-frame deletion mutants were constructed in similar manners by plasmid pK18*mobsacB*-Δ*3-mst* and pK18*mobsacB*-Δ*mfs* using primers listed in Additional file 1: Table S2. For performing sensitivity assays, bacteria growth in LB broth containing 0.3 mM cumene hydroperoxide (CHP), 0.9 mM menadione (MEN), 45 mM H_2_O_2_, 0.4 mM HOCl, 1.5 mM tert-butyl hydroperoxide (*t*-BHP), 5 mM iodoacetamide (IAM), 0.1 µg/ml gentamicin, or 17 µM cadmium chloride (CdCl_2_) was measured according to Helbig et al. [38].

### Cloning, expression, and recombinant protein purification

The genes encoding *C. glutamicum* MsrR (NCgl2679), 3-MST (NCgl2678), MFS (NCgl2680) were amplified using primers listed in Additional file 1: Table S2 by PCR. The amplified DNA fragments were digested and subcloned into similar digested pET28a, pXMJ19, or pXMJ19-His_6_ vectors, obtaining pET28a-*msrR*, pXMJ19-*msrR*, pXMJ19-His_6_-*msrR*, pXMJ19-*3-mst*, and pXMJ19-*mfs*, respectively.

The plasmids pK18*mobsacB*-Δ*msrR*, pK18*mobsacB*-Δ*3*-*mst*, and pK18*mobsacB*-Δ*mfs* were constructed by overlap-PCR [39]. Briefly, primer pairs DMsrR-F1/DMsrR-R1 and DMsrR-F2/DMsrR*-*R2 listed in Additional file 1: Table S2 were used to amplify the 806-bp upstream fragment and the 820-bp downstream fragment of *msrR*, respectively. The primer pair DMsrR-F1/DMsrR*-*R2 was used to fuse the upstream and downstream fragments together by overlap extension PCR [39]. The obtained PCR products were digested with *Eco*RI and *Bam*HI, and cloned into similar digested pK18*mobsacB* to produce pK18*mobsacB*-Δ*msrR*. The knock-out plasmid pK18*mobsacB*-Δ*3-mst* and pK18*mobsacB*-Δ*mfs* were constructed in a similar manner by using the primers listed in Additional file 1: Table S2.

The *lacZY* fusion reporter vectors pK18*mobsacB*-*P*_*msrR*_*::lacZY* and pK18*mobsacB*-*P*_*mfs*_*::lacZY* were obtained by fusion of the *msrR *or *mfs* promoter to the *lacZY* reporter gene via overlap-PCR [40]. Firstly, the primers *P*_*msrR*_-F/*P*_*msrR*_-R and lacZY-F/lacZY-R were used in the first round of PCR to amplify the 232-bp *msrR *promoter DNA fragments (corresponding to nucleotides + 12 to – 220 relative to the translational start codon (ATG) of *msrR* gene) and the *lacZY* DNA fragments, respectively. Secondly, *P*_*msrR*_-F/lacZY-R as primers and the first round PCR products as templates were used to perform the second round of PCR, and the resulting fragments were digested with *Sma*I and *Pst*I, and inserted into similar digested pK18*mobsacB* to obtain the pK18*mobsacB-P*_*msrR*_*::lacZY* fusion construct [29]. A similar process was used to construct pK18*mobsacB-P*_*mfs*_*::lacZY*. Briefly, the 235-bp *mfs* promoter DNA fragments (corresponding to nucleotides + 15 to − 220 relative to the translational start codon (ATG) of *mfs* gene) was amplified with the primers listed in Additional file 1: Table S2 and fused to the *lacZY* reporter genes. The resulting *P*_*mfs*_*::lacZY* was inserted into similar digested pK18*mobsacB.*

For obtaining pK18*mobsacB-P*_*msrRM*_*::lacZY*, 232-bp *msrR* promoter DNA containing mutagenesis sequence of the predicted MsrR binding site (*P*_*msrRM*_) was first directly synthesized by Shanghai Biotechnology Co., Ltd.. Start and stop sites of *P*_*msrRM*_ were the same as those of *P*_*msrR*_ in *P*_*msrR*_*::lacZY*. Then, the resulting 232-bp *P*_*msrRM*_ was fused to a *lacZY* reporter gene*.* Finally, *P*_*msrRM*_*::lacZY* was inserted into similar digested pK18*mobsacB.* A similar process was used to construct pK18*mobsacB-P*_*mfsM*_*::lacZY*. Briefly, 235-bp *mfs* promoter DNA containing a mutagenesis sequence of the predicted MsrR binding site (*P*_*mfsM*_) was directly synthesized and its start and stop sites were the same as those of *P*_*mfs*_ in *P*_*mfs*_*::lacZY*. Then, 235-bp *P*_*mfsM*_ was fused to a *lacZY* reporter gene to obtain *P*_*mfsM*_*::lacZY.* Finally, *P*_*mfsM*_::*lacZY* was inserted into similar digested pK18*mobsacB.*

For complementation or overexpression in *C. glutamicum* strains, pXMJ19 or pXMJ19-His_6_ derivatives were transformed into the corresponding *C. glutamicum* strains by electroporation, and the transformants were selected on 10 µg/ml chloramphenicol and 40 µg/ml nalidixic acid-containing LB agar plates. The transformant’s expression was induced by adding 0.5 mM isopropyl β-d-1-thiogalactopyranoside (IPTG) into medium [40].

To make the cysteine residue at position 62 of MsrR into a serine residue (MsrR:C62S), site-directed mutagenesis was made by two rounds of PCR [41]. In brief, in the first round of PCR, primer pairs DMsrR-F1/MsrR-C62S-R and MsrR-C62S-F/DMsrR-R2 were used to amplify segments 1 and 2, respectively. The second round of PCR was performed by using CMsrR-F/CMsrR*-*R or OMsrR-F/OMsrR*-*R as primers and fragment 1 and fragment 2 as templates to produce the *msrR:C62S* DNA segment. The *msrR:C62S* segment was digested and subcloned into digested pET28a, pXMJ19 or pXMJ19-His_6_ plasmid, obtaining the corresponding plasmids. To express and purify His_6_-tagged recombinant proteins, the pET28a derivatives were transformed into *E. coli* BL21(DE3). Recombinant proteins were purified according to previously described method [40]. Primers used in this study were listed in Additional file 1: Table S2.

The fidelity of all constructs was confirmed by DNA sequencing (Sangon Biotech, Shanghai, China).

### Construction of chromosomal fusion reporter strains and β-galactosidase assay

The *lacZY* fusion reporter plasmids pK18*mobsacB*-*P*_*msrR*_*::lacZY*, pK18*mobsacB*-*P*_*mfs*_*::lacZY*, pK18*mobsacB*-*P*_*msrRM*_*::lacZY*, and pK18*mobsacB*-*P*_*mfsM*_*::lacZY* were transformed into *C. glutamicum* parental strain containing the high copy number of empty plasmid pXMJ19 (the strains were named WT), ∆*msrR* (strains lacking *msrR* gene containing empty pXMJ19) and ∆*msrR*^+^ (Δ*msrR* was complemented with pXMJ19 plasmids carrying the wild-type *msrR* gene) by electroporation, respectively. The introduced pK18*mobsacB* derivatives were integrated into the chromosome using fusion promoter regions homologous to the genome of *C. glutamicum* by single crossover and then the chromosomal WT(*P*_*msrR*_*::lacZY*), ∆*msrR*(*P*_*msrR*_*::lacZY*), ∆*msrR*^+^(*P*_*msrR*_*::lacZY*), WT(*P*_*msrRM*_*::lacZY*), ∆*msrR*(*P*_*msrRM*_*::lacZY*), ∆*msrR*^+^(*P*_*msrRM*_*::lacZY*), WT(*P*_*mfs*_*::lacZY*), ∆*msrR*(*P*_*mfs*_*::lacZY*), ∆*msrR*^+^(*P*_*mfs*_*::lacZY*), WT(*P*_*mfsM*_*::lacZY*), ∆*msrR*(*P*_*mfsM*_*::lacZY*), and ∆*msrR*^+^(*P*_*mfsM*_*::lacZY*) fusion reporter strains were selected by plating on LB agar plates containing 40 µg/ml^−1^ nalidixic acid, 25 µg/ml^−1^ kanamycin, and 10 µg/ml^−1^ chloramphenicol [37]. The resulting strains were grown in LB medium to an optical density at 600 nm of 0.6–0.7 and then treated with different reagents of various concentrations at 30 °C for 30 min. β-galactosidase activities were assayed with o-Nitrophenyl-β-d-Galactopyranoside (ONPG) as the substrate [39]. The standard assay for quantitating the amount of β-galactosidase activity in cells, originally described by Miller for assay of bacterial cultures, involves spectrophotometric measurement of the formation of the yellow chromophore ο-nitrophenol (ONP) as the hydrolytic product of the action of β-galactosidase on the colorless substrate ο-Nitrophenyl β-d-galactopyranoside (ONPG) [42]. All β-galactosidase experiments were performed with at least three independent biological replicates.

### Quantitative real-time polymerase chain reaction (qRT-PCR) analysis

Total RNA was isolated from exponentially growing WT, Δ*msrR* and Δ*msrR*^+^ strains exposed to different toxic agents of indicated concentrations for 30 min using the RNeasy Mini Kit (Qiagen, Hilden, Germany) along with the DNase I Kit (Sigma-Aldrich, Taufkirchen, Germany). Purified RNA was reverse-transcribed with random 9-mer primers and MLV reverse transcriptase (TaKaRa, Dalian, China). Quantitative RT-PCR analysis (7500 Fast Real-Time PCR; Applied Biosystems, Foster City, CA) was performed as described previously [40]. The primers used were listed in Additional file 1: Table S2. To obtain standardization of results, the relative abundance of 16S rRNA was used as the internal standard.

### H_2_O_2_-dependent structural change of MsrR in vivo

The H_2_O_2_-dependent structural change of MsrR and its variant in vivo were determined by a previously reported method [39]. Δ*msrR* (pXMJ19-His_6_-*msrR*) and Δ*msrR* (pXMJ19-His_6_-*msrR:C62S*) strains were cultured in LB containing 0.5 mM IPTG, 10 µg/ml chloramphenicol, and 40 µg/ml nalidixic acid at 30 °C. Cells were grown to mid-exponential phase and split into 100 ml aliquots for H_2_O_2_ treatment (0–30 mM, 60 min). The treated samples were harvested immediately by centrifugation, broken through ultrasound on ice, and then crude cell lysates were centrifuged. Obtained supernatants were subjected to nonreducing sodium dodecyl sulfate–polyacrylamide gel electrophoresis (SDS-PAGE) or reducing SDS-PAGE, and the structural properties of MsrR and its variant were visualized by immunoblotting using the anti-His antibody.

### Electrophoretic mobility shift assay (EMSA)

EMSA was performed using the method of Si et al. [30]. Briefly, a 162-bp *msrR* promoter sequence [*P*_*msrR*_; corresponding to nucleotides − 154 to + 8 relative to the translational start codon (GTG) of the *cssR* ORF] containing the predicted MsrR binding site was amplified using primer pair EMsrR-F/EMsrR-R (Additional file 1: Table S2). The binding reaction mixture (20 μl) contained 10 mM Tris–HCl (pH 7.4), 5 mM MgCl_2_, 50 mM KCl, 5% glycerol, 0.1% Nonidet P 40 (NP40), 1 μg poly(dI:dC), 0–60 nM of MsrR, and 40 ng *P*_*msrR*_. 162-bp DNA fragments amplified from MsrR ORF (40 ng) instead of *P*_*msrR*_ were used as a negative control. A 162-bp EMSA promoter DNA containing the mutated sequence of the predicted MsrR-binding site and having the same start and stop sites as *P*_*msrR*_ (*P*_*msrRM*_) was directly synthesized by Shanghai Biotechnology Co., Ltd.. After the binding reaction mixture was incubated at room temperature for 30 min, the mixture was subjected to electrophoresis on 8% nondenaturing polyacrylamide gel made with 10 mM Tris buffer containing 50 mM KCl, 5 mM MgCl_2_ and 10% glycero1 in 0.5× TBE electrophoresis buffer [50 mM Tris, 41.5 mM borate (pH 8.0), 10 mM Na_2_EDTA.H_2_O], and stained either with a 10,000-fold diluted Synergy Brand (SYBR) Gold nucleic acid staining solution (Molecular Probes) or GelRed™ and photographed. The DNA bands were visualized with UV light at 254 nm.

The reversibility of the loss of binding due to oxidation was tested as follows. H_2_O_2_ was added to MsrR solution to a final concentration of 10 mM, immediately aliquots were taken and incubated with 40 ng *P*_*msrR*_ for EMSA. In the next step, dithiothreitol (DTT) was added to the H_2_O_2_-treated MsrR solutions to a final concentration of 50 mM, and again aliquots were taken for EMSA. All aliquots were incubated in binding buffer with 40 ng *P*_*msrR*_ for 30 min at room temperature and separated on an 8% nondenaturing polyacrylamide gel and the gel was stained using SYBR Gold nucleic acid staining solution.

For the determination of apparent *K*_*D*_ values, increasing concentrations of the MsrR (0–100 nM) were incubated for 30 min at room temperature with 40 ng *P*_*msrR*_. The samples were applied onto an 8% native polyacrylamide gel and separated at 180 V for 1 h on ice. The gels stained with GelRed™ and photographed were quantified using ImageQuant software (GE Healthcare), and the percentage of shifted DNA was calculated. These values were plotted against the MsrR concentration in log_10_ scale, and a sigmoidal fit was performed using GraphPad Prism software (GraphPad Software, San Diego California USA), considering the error bars as well as 0 and 100% shifted DNA as asymptotes, the turning point of the curve was defined as the apparent *K*_*D*_ value. All determinations were performed in triplicate.

### Western blot analysis

Western blot analysis was conducted as previously described [29]. The cytosolic RNA polymerase β (RNA polβ) was used as a loading control as in our previous study [29].

### Statistical analysis

Statistical analyses of survival rate, transcription level and protein level were determined with paired two-tailed Student’s t-test. GraphPad Prism Software was used to carry out statistical analyses (GraphPad Software, San Diego California USA).

## Results and discussion

### The Δ*msrR C. glutamicum* strain showed reduced sensitivity to challenge by oxidants, antibiotics, heavy metals, and alkylating agents

**Fig. 1:**
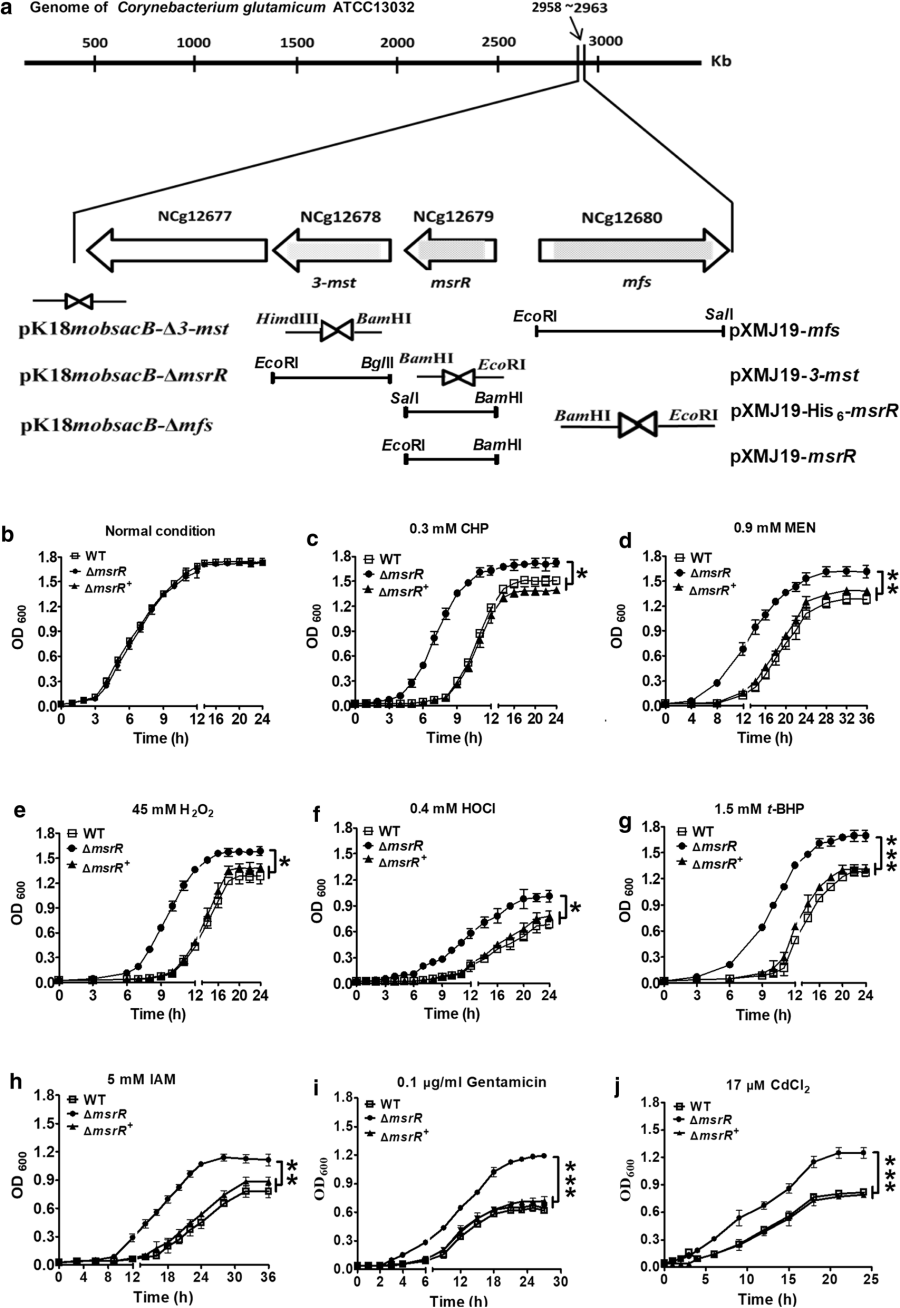
MsrR was required for optimal growth under various stress. **a** Physical map of the *msrR*-*3-mst-ncgl2677* genetic cluster and *mfs* gene in *Corynebacterium glutamicum* (upper panel) and construction of plasmids for gene disruption (pK18*mobsacB* derivatives) and complementation (pXMJ19 derivatives) (lower panel). Open reading frames (ORFs) were marked by open arrows, and the deleted regions were in gray. The restriction sites were indicated. *msrR*, *3-mst*, and *mfs* represented *ncgl2679*, *ncgl2678*, and *ncgl2680* genes, encoding multiple stress resistance regulator, 3-Mercaptopyruvate sulfurtransferase, and a major facilitator superfamily protein, respectively. **b** Growth of the indicated three strains in LB broth without stress was used as control. **c–j** Growth of indicated strains in LB broth with 0.3 mM cumene hydroperoxide (CHP), 0.9 mM menadione (MEN), 45 mM hydrogen peroxide (H_2_O_2_), 0.4 mM hypochlorous acid (HOCl), 1.5 mM tert-butyl hydroperoxide (*t*-BHP), 5 mM iodoacetamide (IAM), 0.1 µg/ml gentamicin, or 17 µM cadmium chloride (CdCl_2_), respectively. Data show the averages of three independent experiments, and error bars indicated the SDs from three independent experiments. ***P ≤ 0.001; **P ≤ 0.01; *P ≤ 0.05

The 723-bp *C. glutamicum ncgl2679* gene is located from bp 2,960,466 to 2,961,188 (Fig. 1a, upper panel) and encodes a hypothetical transcriptional regulator consisting of 240 amino acid residues with a molecular mass of 26.2 kDa. The putative protein product, which contains a helix-turn-helix motif, shares similarity with XRE (xenobiotic response element) family transcription factors from *Corynebacterium crudilactis*, *Corynebacterium efficiens*, *Corynebacterium callunae*, *Corynebacterium epidermidicanis*, and *Corynebacterium minutissimum* (80%, 68%, 64%, 42%, and 40% amino acid sequence identity, respectively) (Additional file 1: Figure S1). A recent study showed that the transcriptional regulator SrtR, an XRE family member, is involved in oxidative and high temperature stress tolerance [19]. This finding prompted us to examine whether NCgl2679 plays a role in protecting the soil bacterium *C. glutamicum* from various stresses. The functions of NCgl2679 were identified by gene disruption and complementation (Fig. 1a, lower panel). Growth analysis of different *C. glutamicum* strains on LB medium in the absence of stress revealed that the wild-type *C. glutamicum* strain (WT, *C. glutamicum* transformed with the empty plasmid pXMJ19), the Δ*ncgl2679* mutant strain (the *ncgl2679* deletion mutant expressing pXMJ19) and the Δ*ncgl2679*^+^ strain (the *ncgl2679* deletion mutant expressing the wild-type *ncgl2679* gene in the shuttle vector pXMJ19) showed almost the same growth rates (Fig. 1b). However, the growth of the WT strain in LB medium containing oxidants, alkylating agents, antibiotics, or heavy metals was markedly inhibited relative to the growth of the Δ*ncgl2679* mutant strain (Fig. 1c–j). The complementary strain Δ*ncgl2679*^+^ exhibited a growth rate equivalent to that of the wild-type strain under various stresses, consistent with a previous evaluation of XREs under stress [19]. These results indicated that NCgl2679 is involved in the resistance of *C. glutamicum* to various stresses. Thus, we named NCgl2679 multiple stress response regulator (MsrR).

### MsrR negatively regulates expression of the divergently oriented genes *mfs* and *msrR*-*3-mst*

In the *C. glutamicum* genome, *msrR* (*ncgl2679*) is organized in a putative operon with *ncgl2678* and *ncgl2677*, which were shown to be co-transcribed by reverse transcription PCR (Additional file 1: Figure S2). Further downstream from *ncgl2679* is the *ncgl2680* gene, which was annotated as the multidrug efflux protein MFS. The *mfs* and *msrR* genes are oriented in opposite directions*.* By bioinformatics molecular analysis, two putative overlapping and divergent promoter sequences in the intergenic region between the start codons of *mfs* and *msrR* were found (Additional file 1: Figure S3), and one of these promoter sequences was found to be located upstream of the *msrR* gene. Neighboring *mfs* is a putative − 10 and − 35 promoter sequence, which was found to be the *mfs* promoter.

**Fig. 2:**
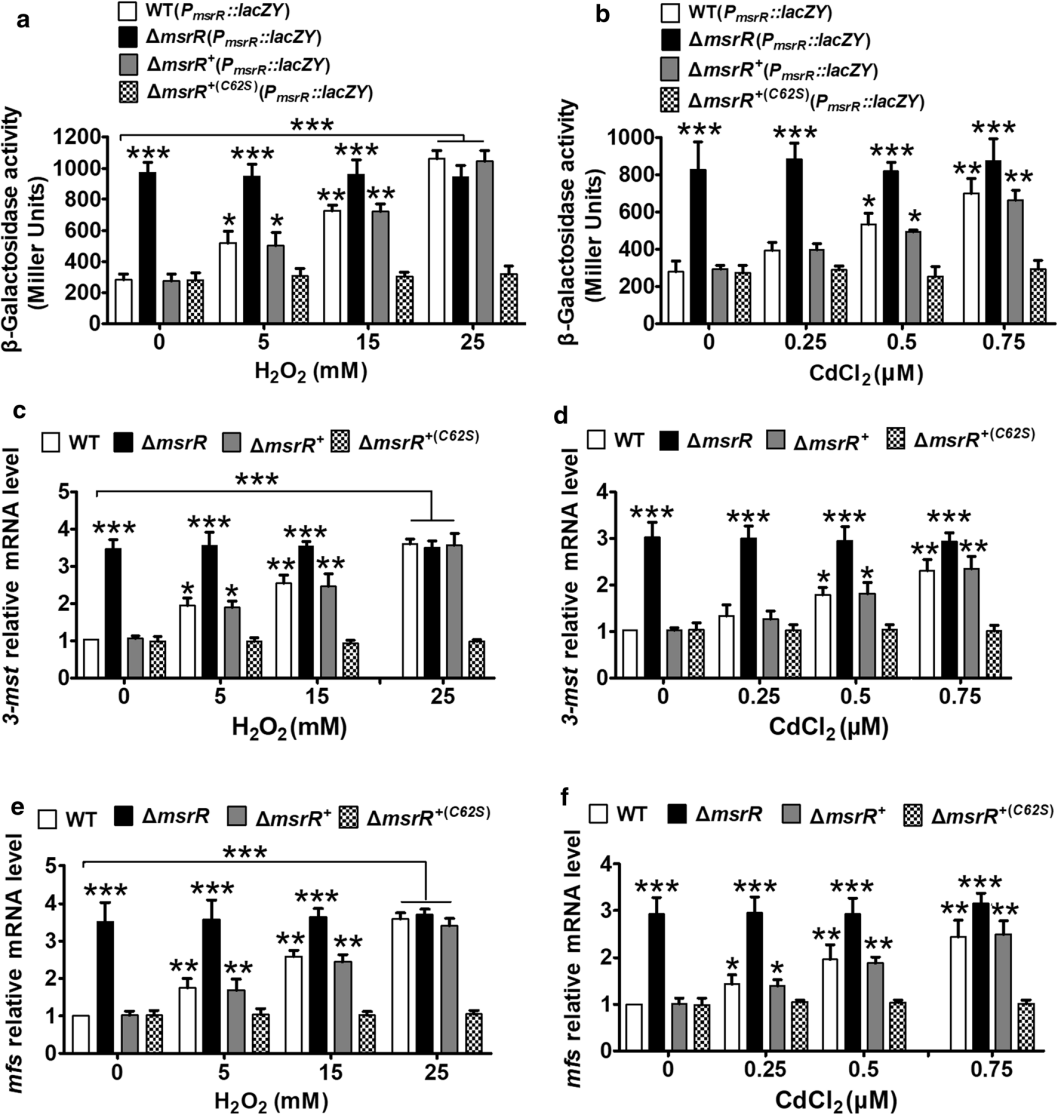
Negative regulation of *msrR*-*3*-*mst* and *mfs* expressions by MsrR. **a**, **b** β-galactosidase analysis of the *msrR* promoter activity was performed using the transcriptional *P*_*msrR*_*::lacZY* chromosomal fusion reporter expressed in indicated strains exposed to H_2_O_2_ and CdCl_2_. **c**–**f** qRT-PCR assay was performed to analyze the expression of *3-mst* and *mfs* in indicated strains exposed to H_2_O_2_ and CdCl_2_. The mRNA levels were presented relative to the value obtained from WT cells without treatment. Relative transcript levels of WT strains without stress treatment were set at a value of 1.0. Data show the averages of three independent experiments, and error bars indicated the SDs from three independent experiments. ***P ≤ 0.001; **P ≤ 0.01; *P ≤ 0.05

On the basis of bioinformatics analysis, a putative MsrR-binding site in the putative overlapping, divergent promoters of the *msrR*-*ncgl2678* locus and *mfs* gene was found (Additional file 1: Figure S3). Thus, we speculated that MsrR negatively regulates the *msrR*-*ncgl2678-ncgl2677* locus and represses transcription of the adjacent, oppositely oriented *mfs* gene. To verify this speculation, *msrR*, *ncgl2678* and *mfs* transcription levels in the WT, Δ*msrR* mutant, and Δ*msrR*^+^ strains were analyzed by qRT-PCR and determination of the *lac*Z*Y* activity of the chromosomal promoter fusion reporter. Notably, to study the expression of *msrR* in the Δ*msrR* mutant strain by qRT-PCR, a 104-bp *msrR* transcript (corresponding to nucleotides + 1 to + 104 relative to the translational start codon (GTG) of the *msrR* gene) was amplified from the remaining *msrR* ORF in the Δ*msrR* mutant strain with the primers QmsrR-F and QmsrR-R (Additional file 1: Figure S4). As expected, *msrR*, *ncgl2678* and *mfs* transcription levels in the Δ*msrR* mutant strain were obviously higher than those in the WT and Δ*msrR*^+^ strains (Fig. 2 and Additional file 1: Figure S5). These results indicated that MsrR negatively controls the expression of NCgl2678, MFS, and its structural gene.

**Fig. 3:**
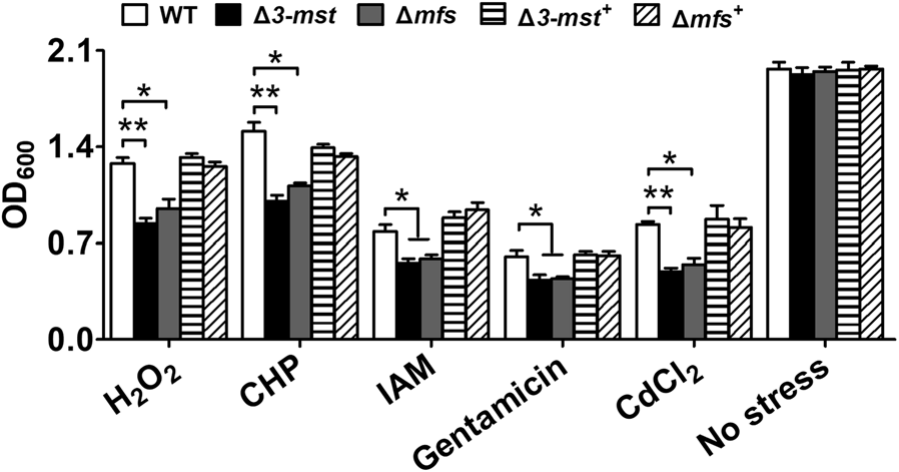
3-MST and MFS were involved in oxidative stress resistance. Effect of deletion of *3-mst* and *mfs* genes on various stress resistance. The growth (OD_600_) of the indicated strains after over 24 h at 30 °C in LB medium containing various stress was recorded. Data show the averages of three independent experiments, and error bars indicated the SDs from three independent experiments. **P ≤ 0.01; *P ≤ 0.05

*ncgl2678*, which was annotated as 3-mercaptopyruvate sulfurtransferase (3-MST), is mainly responsible for hydrogen sulfide (H_2_S) production [43]. Previous studies found that H_2_S made by nonsulfur bacteria alleviates oxidative stress imposed by diverse stresses through increasing levels of intracellular antioxidants, including glutathione (GSH); antioxidant enzymes; and glutamate uptake [44, 45]. This finding suggests that the absence of *3-mst* probably cause the decrease of H_2_S content, which in turn reduction of the antioxidant capacity of *C. glutamicum* strains. In addition, many reports have revealed that cells expressing MFS can excrete various poisons [46, 47], suggesting that *C. glutamicum* MFS is also important for resistance to diverse stresses. Thus, the functions of *3-mst* and *mfs* were identified by gene disruption and complementation with *C. glutamicum* (Fig. 1a, lower panel). As shown in Fig. 3, while deletion of *3-mst* or *mfs* did not affect bacterial growth under normal conditions, compared to the WT strain, the Δ*3-mst* and Δ*mfs* mutant strains devoid of *3-mst* or *mfs*, respectively, exhibited obvious growth inhibition under challenge with various diverse stresses. The growth of *3*-*mst* or *mfs* deletion mutant strains under diverse stresses was restored to a level similar to that of the WT strain by transformation with the plasmid-encoded wild type *3*-*mst* or *mfs* gene (Δ*3*-*mst*^+^or Δ*mfs*^+^), in agreement with the results of Li et al. regarding MST [48] (Fig. 3).

### Expression of *msrR*, *3-mst* and *mfs* was induced by oxidative stress via MsrR

Previous studies revealed that the transcriptional activation of target genes controlled by XREs is mediated by xenobiotics, which act as inducers [49, 50]. The mechanism by which various xenobiotics act as inducers and affect the conformation of XREs is a key feature for induction activity. Thus, these studies, combined with the above finding that MsrR is involved in tolerance to various stresses, led us to investigate whether MsrR participates in the induction of its own gene and the *3-mst* and *mfs* genes by xenobiotics. For simplicity, we used H_2_O_2_ and CdCl_2_ as inducers in the following experiments. As shown in Fig. 2a and Additional file 1: Figure S5c, in the absence of H_2_O_2_, the Δ*msrR* strain had significantly higher *msrR* and *mfs* expression levels than the WT and Δ*msrR*^+^ strains, whereas the *lacZY* activities of *msrR* and *mfs* in the WT strain exposed to H_2_O_2_ were obviously higher than those in the untreated-H_2_O_2_ WT strain. The addition of H_2_O_2_ did not change the *lacZY* activities of *msrR* or *mfs* in the Δ*msrR* strain, which were maintained at the same levels observed in the Δ*msrR* strain without H_2_O_2_ treatment. Moreover, analysis of the *lacZY* activities showed a dose-dependent change in expression in the WT and Δ*msrR*^+^ strains in response to H_2_O_2_ (Fig. 2a and Additional file 1: Figure S5c). A similar regulatory pattern of *msrR*, *3-mst* or *mfs* by MsrR was also observed at the mRNA transcriptional level by qRT-PCR analysis (Fig. 2c, e and Additional file 1: Figure S5a). These results clearly demonstrated that *msrR*, *3-mst* and *mfs* were upregulated in response to increasing H_2_O_2_ concentration, indicating that oxidation inhibited the DNA binding of MsrR, inducing the expression of its own gene and the *3-mst* and *mfs* genes. This derepression of *msrR*, *3-mst* and *mfs* transcription by CdCl_2_ was mediated via MsrR in a matter similar to that of H_2_O_2_ (Fig. 2b, d, f and Additional file 1: Figure S5b, d).

### The ability of MsrR to bind the intergenic region between* msrR *and *mfs* was reversibly inhibited by ROS

**Fig. 4:**
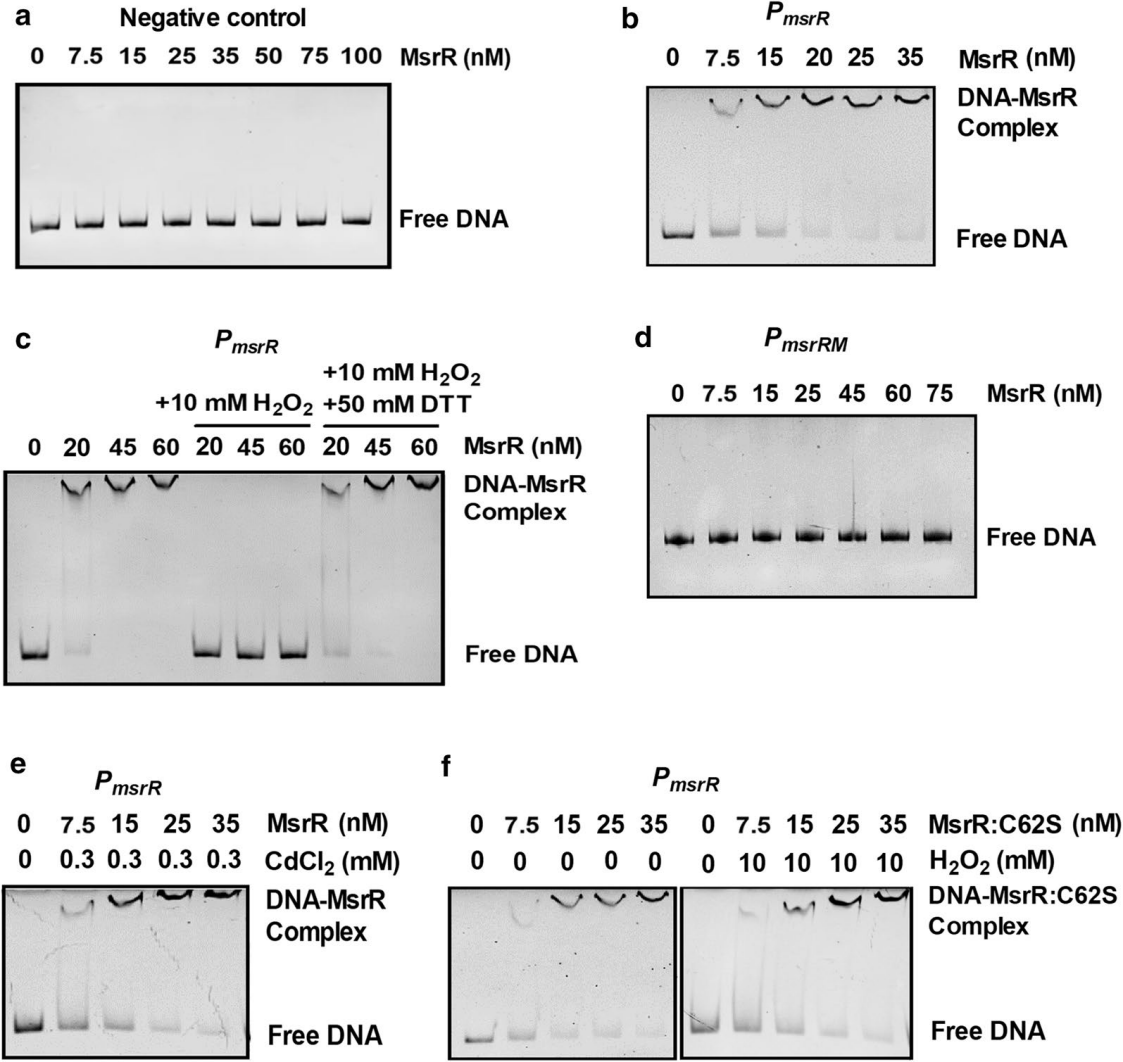
Reversible inhibition of the DNA binding activity of MsrR by H_2_O_2_ and role of Cysteine residue. **a** The interaction between His_6_-MsrR and DNA fragments amplified from MsrR’s ORF. **b** The interaction between His_6_-MsrR and the promoter fragment in the intergenic region between *msrR* and *mfs* (named *P*_*msrR*_). **c** Inhibition of the DNA binding activity of MsrR by H_2_O_2_ and reversal of the inhibition by DTT. MsrR was prepared in three different concentrations, and aliquots were taken for EMSAs (control). Then H_2_O_2_ was added to the binding reaction mixture to a final concentration of 10 mM, and aliquots were taken for EMSA. In the next step DTT (a final concentration of 50 mM) was added to 10 mM H_2_O_2_-containing binding reaction mixture, and aliquots were taken for EMSAs. All aliquots were incubated in binding buffer, pH 8.0, with 40 ng *P*_*msrR*_ and then separated on an 8% native polyacrylamide gel. **d** The interaction between His_6_-MsrR and the promoter mutating the predicted MsrR binding region (*P*_*msrRM*_). **e** CdCl_2_ was added to the binding reaction mixture to a final concentration of 0.3 mM, and the interaction between His_6_-MsrR and *P*_*msrR*_ was performed. **f** The interaction between the mutated derivatives MsrR:C62S and *P*_*msrR*_ in the absence (left panel) or presence (right left) of 10 mM H_2_O_2_. Results were obtained in three independent experiments, and data show one representative experiment done in triplicate

To determine whether MsrR directly regulates its own transcription and the transcription of 3-MST and MFS, we examined the interaction between purified MsrR and a DNA promoter fragment in the intergenic region between *msrR* and *mfs* (named *P*_*msrR*_) using EMSA. Incubation of *P*_*msrR*_ with His_6_-MsrR caused a clear delay in promoter DNA migration, and *P*_*msrR*_ migrated in a manner dependent on the concentration of His_6_-MsrR (Fig. 4b and Additional file 1: Figure S6b). The apparent *K*_*D*_ value for *P*_*msrR*_ was about 17 nM MsrR (Additional file 1: Figure S7a), which is within the range found for other transcriptional regulators [33]. Moreover, this effect was specific because the combination of His_6_-MsrR and DNA fragments amplified from the MsrR ORF did not delay migration (Fig. 4a and Additional file 1: Figure S6a). However, the binding of His_6_-MsrR to *P*_*msrR*_ was prevented by the addition of 10 mM H_2_O_2_ (Fig. 4c and Additional file 1: Figure S6c)*.* Importantly, the impaired DNA-binding activity of His_6_-MsrR by H_2_O_2_ could be restored via the addition of an excess of the reducing agent DTT (50 mM), indicating that the effects of oxidation and reduction on the DNA-binding activity of MsrR were reversible. (Fig. 4c and Additional file 1: Figure S6c). Mutations in the predicted MsrR-binding site (a 162-bp EMSA promoter DNA contained the mutated sequence of the predicted MsrR-binding site (*P*_*msrRM*_), which had the same start and stop sites as *P*_*msrR*_) (Additional file 1: Figure S3) disrupted the formation of DNA–protein complexes (Fig. 4d and Additional file 1: Figure S6d), and promoter DNA mutations in the predicted MsrR-binding site (a 232-bp DNA fragment contained the mutated sequence of the predicted MsrR-binding site for *lacZY* activity, which had the same start and stop sites as a 232-bp DNA fragment on *P*_*msrR*_*::lacZY*; a 235-bp DNA fragment contained the mutated sequence of the predicted MsrR-binding site for *lacZY* activity, which had the same start and stop sites as a 235-bp DNA fragment on *P*_*mfs*_*::lacZY*) caused extremely high *P*_*msrRM*_*::lacZY* and *P*_*mfsM*_*::lacZY* activities in the WT and Δ*msrR*^+^ strains, similar to those in the Δ*msrR* mutant strain (Additional file 1: Figure S8), further indicating the recognition of DNA elements by MsrR. Interestingly, the addition of CdCl_2_ did not induce the dissociation of MsrR from *P*_*msrR*_, inconsistent with the finding that derepression of *msrR* transcription by CdCl_2_ was mediated via MsrR in vivo (Fig. 4e and Additional file 1: Figure S6e)*.* Combined with the discovery that expression of *msrR* was affected by H_2_O_2_ (Fig. 2), we speculated that this was related to CdCl_2_-mediated perturbation of the electron transfer chain, resulting in the formation of ROS in vivo, which inactivated XRE DNA-binding activity by the oxidation of cysteine residues [51, 52]. In fact, many studies have reported that the most potent xenobiotics, including oxidants, alkylating agents, antibiotics, and heavy metals, can generate ROS by redox cycling to produce oxidative stress inside bacteria [1, 51–56]. Thus, we speculated that MsrR does not directly sense ligands such as CdCl_2_, gentamicin, MEN and IAM.

Together, these results show that MsrR specifically recognized operators and then directly bound the *msrR* and *mfs* intergenic region in a sequence-specific manner. Upon exposure to oxidative stress, MsrR was inhibited by changes in conformation caused by ROS and released from the promoter, leading to the upregulation of target genes.

### Oxidation promoted MsrR dimerization and inaction

**Fig. 5:**
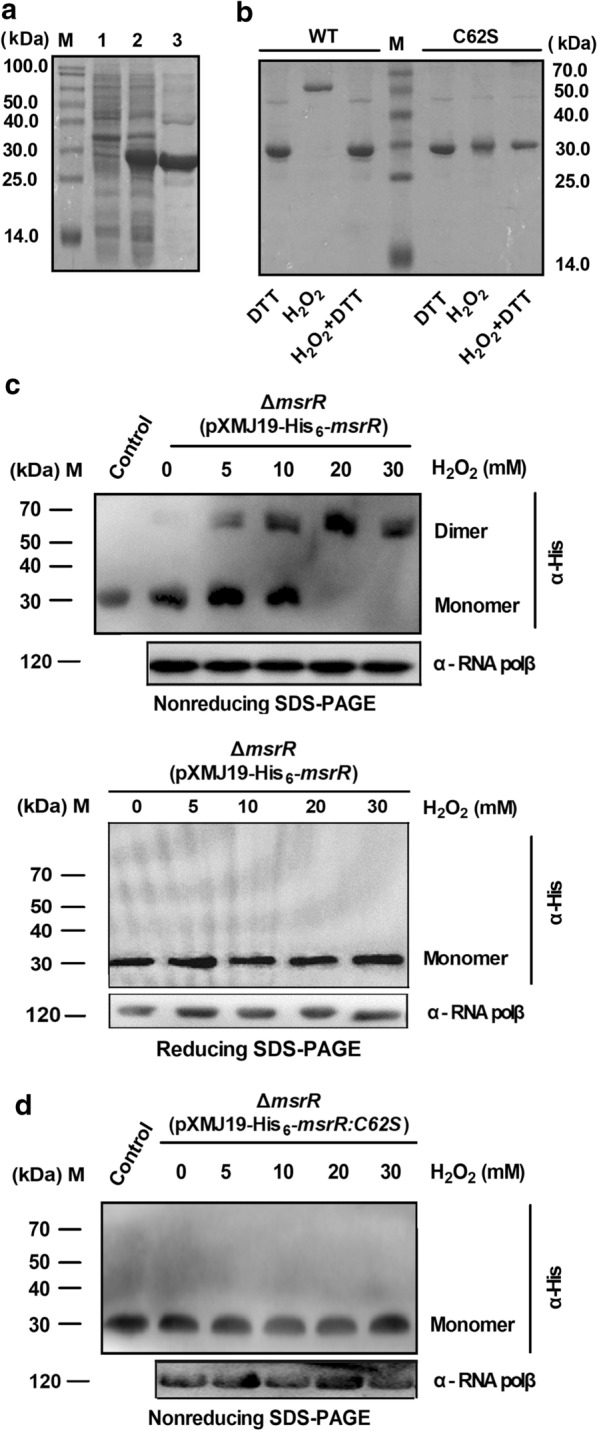
Redox response of MsrR in vitro and in vivo. **a** Nonreducing SDS-PAGE analysis of proteins expressed in *E. coli* containing pET28a-*msrR* plasmid. M, broad-range protein marker; lane 1, crude extract (5 μg) without IPTG induction; lane 2, crude extract (5 μg) with induction; lane 3 purified His_6_-MsrR protein. **b** Redox response of MsrR and its variant detected by nonreducing SDS-PAGE. 15 μM proteins treated with 50 mM DTT were further incubated with or without 50 μM H_2_O_2_, or 50 μM H_2_O_2_ and 50 mM DTT, and then samples were separated by 15% nonreducing SDS-PAGE. **c**, **d** Oxidative stress-dependent structural changes of relevant MsrR in vivo. Proteins extracted from cells exposed to different concentrations of H_2_O_2_ for 30 min were resolved on nonreducing or reducing SDS-PAGE, and analyzed with Western blotting by using the anti-His antibody. RNA polymerase β (RNA polβ) was used as a loading control. Similar results were obtained in three independent experiments, and data show one representative experiment done in triplicate

Many redox-sensitive regulators, such as RosR, CosR, and OhsR, exist as homodimers via intersubunit disulfide bonds upon oxidation [29, 30, 33]. The amino acid sequence of MsrR shows that it contains one cysteine residue at position 62 (Additional file 1: Figure S1). Thus, we thought it might share a similar oxidation-sensing mechanism and that MsrR is oxidized to form an intersubunit disulfide-containing dimer. As shown in Fig. 5a, nonreducing SDS-PAGE showed that the native MsrR protein was monomeric with an apparent MW of approximately 30 kDa, corresponding well to the molecular mass of MsrR deduced from its amino acid sequence, while MsrR incubated with H_2_O_2_ migrated as a band of approximately 60 kDa, as judged by its behavior on 15% nonreducing SDS-PAGE, which corresponded to MsrR in its dimeric form. This dimeric formation was reversed by an excess of DTT (Fig. 5b). Moreover, dimers of H_2_O_2_-treated MsrR:C62S were not observed. These results suggested that the formation of dimeric MsrR occurs via a disulfide bond between MsrR proteins.

To further examine whether the formation of MsrR dimers can be induced under H_2_O_2_ treatment in vivo, we treated cells of the Δ*msrR* (pXMJ19-His_6_-*msrR*) and Δ*msrR* (pXMJ19-His_6_-*msrR:C62S*) strains with H_2_O_2_ at various concentrations and probed the forms of MsrR by immunoblotting with anti-His antibody after nonreducing SDS-PAGE separation (Fig. 5c, d; Additional file 1: Figure S9). Under normal conditions (no stress), MsrR in the Δ*msrR* (pXMJ19-His_6_-*msrR*) strain existed as monomers, but upon exposure to different concentration of H_2_O_2_, the monomeric form changed into an intermolecular disulfide bond-containing dimeric form (Fig. 5c, upper panel and Additional file 1: Figure S9a, upper panel). The dimeric form completely disappeared on reducing SDS-PAGE, indicating that dimeric MsrR in vivo could be also reversed, which was consistent with the results in vitro (Fig. 5c, lower panel and Additional file 1: Figure S9a, lower panel). However, whether under H_2_O_2_ treatment or not, MsrR in the Δ*msrR* (pXMJ19-His_6_-*msrR:C62S*) strain existed in a monomeric form (Fig. 5d and Additional file 1: Figure S9b). These results indicated that H_2_O_2_ causes a structural change in MsrR and that Cys62 is responsible for the morphological changes in MsrR observed under H_2_O_2_ treatment.

### Inactivation of the DNA binding of MsrR by ROS is dependent on the oxidation state of Cys62

The reduction and oxidation of cysteine residues is involved in the control of ROS-sensing sensor activity [10]. It would be interesting to know whether Cys62 of MsrR plays an important role in the H_2_O_2_-sensing and transcription mechanisms of MsrR. Thus, the ability of the MsrR:C62S variant to suppress *msrR*, *3-mst* and *mfs* expression in response to H_2_O_2_ was evaluated in the Δ*msrR* strain using promoter *lac*Z*Y* activity and qRT-PCR analysis. Analysis of the transcriptional levels revealed that Δ*msrR*^+(*C62S*)^ (the Δ*msrR* strain containing the pXMJ19-*msrR:C62S* plasmid) inhibited *msrR*, *3-mst* and *mfs* expression under H_2_O_2_ treatment conditions to equal degrees, similar to that in the untreated-H_2_O_2_ WT strain, indicating that Cys62 plays a role in the dissociation of MsrR from the promoter under H_2_O_2_ treatment conditions (Fig. 2 and Additional file 1: Figure S5**)**.

To further probe whether Cys62 is responsible for the observed dissociation of MsrR under oxidation, MsrR:C62S was used instead of WT MsrR to perform the EMSA experiment. As shown in Fig. 4f and Additional file 1: Figure S6f, in the presence or absence of 10 mM H_2_O_2_, MsrR:C62S still exhibited obviously retarded mobility. Although its affinity constant for *P*_*msrR*_ (*K*_*D*_ = 23.08) was slightly high than that of MsrR, MsrR:C62S behaved high similarly to MsrR without H_2_O_2_ condition (Additional file 1: Figure S7b). These results mean that oxidation of Cys62 was important for inhibition of DNA binding by H_2_O_2_. The above results further showed that the inhibition of DNA binding by H_2_O_2_ was caused by the oxidation of cysteine residue.

## Conclusions

Thiol-based redox-sensing regulators are recognized as an efficient way to combat diverse ROS-inducing stress conditions and enhance the survival of bacteria under oxidative stress. The XRE family is involved in the control of the response to environmental stress, but the functions of XREs related to oxidative stress tolerance, especially their antioxidative molecular mechanisms, are very rarely reported. In this study, we found a MsrR-binding site in the intergenic region between two divergent gene clusters, *msrR*-*3-mst* and *mfs*. β-galactosidase activity assay and qRT-PCR analysis showed that MsrR is indeed negatively autoregulated and also negatively controls the adjacent *3-mst* and *mfs*. In vivo*,* expression of *msrR* is induced by H_2_O_2_ and CdCl_2_, and the *msrR*-deleted (Δ*msrR*) mutant displays increased resistance to H_2_O_2_ and CdCl_2_. However, EMSA experiment shows the ability of MsrR to bind the promoter DNA is inhibited by H_2_O_2_ but not CdCl_2_. Many studies reported that the most potent xenobiotics, including oxidants, alkylating agents, antibiotics, or heavy metals, are capable of generating ROS by redox-cycling to produce oxidative stress inside bacteria [51–56]. Thus, CdCl_2_ might contribute indirectly to ROS production, thereby leading to the derepression of the MsrR operon. Considering high gentamicin- and alkylating agents-resistant phenotype of Δ*msrR* strains, we speculated that antibiotics and alkylating agents might also mediate the DNA binding of MsrR with a mechanism similar to CdCl_2_. We further verified that the XRE-type regulatory MsrR senses and responds to oxidative stress by a derepression of the *msrR*, *3-mst* and *mfs* genes via intermolecular disulfide formation. Mutational analysis of the sole cysteine in MsrR showed that Cys62 is critical for inactivation of the DNA binding of MsrR, distinguishing it from previously discovered stress response properties of XREs in eukaryotes. On the contrary, the regulatory mechanism of MsrR is similar to those of the ROS sensors OxyR, PerR, and OhrR, which are activated or inhibited by changes in conformation caused by cysteines oxidation.

The XRE family is the second most common family of regulators in bacteria, only four members of which have been reported in previous researches, including *S. suis* SrtR [19], *S. aureus* XdrA (XRE-like DNA-binding regulator, A) [21], *R. etli* RHE-CH00371 [22], and *C. aurantiacus* MltR (MmyB-like transcription regulator) [23]. Except for SrtR, no obvious effect on oxidative stress resistance for any of the previously studied examples has been reported so far. *S. aureus* XdrA is shown to play an important role in the β-lactam stress response. Expression of *R. etli* RHE-CH00371 is reported to be down-regulated in an H_2_O_2_-sensitive *R. etli* mutant. *C. aurantiacus* MltR is described as being involved in the regulation of antibiotic biosynthesis and thus represents an example for a rather specialized XRE-type regulator. Sequence analysis clearly indicates that the similarity between MsrR and the XREs of bacteria mentioned above is very low, and Cys62 of MsrR is not very conserved (Figure S1b–e), which only appears in position 66 of *S. suis* SrtR and 55 of *S. aureus* XdrA. The result is consistent with the previous report that the XRE family contains more than 35,000 proteins and more than 70 structures are available [23]. We suggested that differences in structure may cause versatile features and regulatory mechanisms. It is important to point out, despite their low sequence similarity to MsrR (about 30% identity), we thought *S. suis* SrtR and *S. aureus* XdrA might share an oxidation-sensing mechanism as they not only contain the cysteine presumed to serve for oxidation sensing in a relatively conserved position, but they confer resistance to oxidant and β-lactam, respectively, which is similar to MsrR. Combining a phenomenon that β-lactam antibiotics, such as penicillin, can also generate ROS by redox-cycling to produce oxidative stress inside bacteria [55], we speculate that *S. suis* SrtR and *S. aureus* XdrA act as a transcriptional sensor via cysteine oxidation-based thiol modifications. Thus, our results provided, for the first time, insight into a new regulatory mechanism adopted by an XRE protein in which DNA-binding ability is regulated by the oxidation of a cysteine residue in the MsrR protein in response to oxidants but not directly bound ligands, such as antibiotics, heavy metals, and alkylating agents. Our data further confirmed the results of Hu et al. showing a member of the XRE family of transcriptional regulators responsible for oxidant tolerance in bacteria [19], facilitating understanding of antioxidant mechanisms in bacteria and providing initial insight into the molecular mechanisms of XREs involved in oxidative stress tolerance. In addition, MsrR is found to be widely distributed in several species of the genera *Corynebacterium*, such as *C. crudilactis*, *C. efficiens*, *C. callunae*, *C. epidermidicanis*, and *C. minutissimum*. Therefore, our study on the regulatory mechanism of MsrR may lead to a better understanding of the stress response mechanisms of these species. Together, our data show that *C. glutamicum* MsrR acts as a thiol-based redox sensor and, with 3-MST and MFS, comprises an important pathway for protection against oxidative stress.

## Supplementary information

**Additional file 1: Table S1.** Bacterial strains and plasmids used in this study. **Table S2.** Primers used in this study. **Figure S1.** Multiple sequence alignment of MsrR with XREs from other organisms. **Figure S2.** Assays for the *ncgl2679*-*ncgl2678*-*ncgl2677* co-transcription by reverse transcription PCR. **Figure S3.** Detailed genetic maps of the regulatory region of MsrR. **Figure S4.** 104-bp *msrR* transcript (from the translational start codon (GTG) of *msrR* gene to 104th nucleotide) was amplified from the remaining *msrR* ORF (Open Reading Frame) in Δ*msrR* mutant with primers QmsrR-F and QmsrR-R. **Figure S5.** Negative regulation of *msrR* and *mfs* by MsrR. **Figure S6.** Reversible inhibition of the DNA binding activity of MsrR by H_2_O_2_ and role of Cysteine residue. **Figure S7.** Determination of the apparent *K*_*D*_ values of MsrR and MsrR:C62S for *P*_*msrR*_. **Figure S8.** Mutations in the predicted MsrR binding site derepressed the *msrR* expression. **Figure S9.** Oxidative stress-dependent structural changes of relevant MsrR in vivo.

## Data Availability

All the data generated or analyzed during this study are included in the manuscript and its additional file.

## Abbreviations

ROS: Reactive oxygen species
CHP: Cumene hydroperoxide
*t*-BHP: *t*-Butyl hydroperoxide
H_2_O_2_: Hydrogen peroxide
MSH: Mycothiol
GSH: Tripeptide glutathione
MEN: Menadione
DMPD: N,N-dimethyl-p-phenylenediamine
ONPG: ο-Nitrophenyl β-d-galactopyranoside
DTT: Dithiothreitol
3-MST: 3 Mercaptopyruvate sulfurtransferase
XRE: Xenobiotic response element
HOCl: Hypochlorous acid
