# Supplementary material for: MsrR is a thiol-based oxidation-sensing regulator of the XRE family that modulates C. glutamicum oxidative stress resistance
Source: Microb Cell Fact. 2020 Oct 2;19:189. doi: 10.1186/s12934-020-01444-8 (PMC7532634; doi:10.1186/s12934-020-01444-8)
Supplement: Supplementary file 1 — Additional file 1: Table S1. Bacterial strains and plasmids used in this study. Table S2. Primers used in this study. Figure S1. Multiple sequence alignment of MsrR with XREs from other organisms. Figure S2. Assays for the ncgl2679-ncgl2678-ncgl2677 co-transcription by reverse transcription PCR. Figure S3. Detailed genetic maps of the regulatory region of MsrR. Figure S4. 104-bp msrR transcript (from the translational start codon (GTG) of msrR gene to 104th nucleotide) was amplified from the remaining msrR ORF (Open Reading Frame) in ΔmsrR mutant with primers QmsrR-F and QmsrR-R. Figure S5. Negative regulation of msrR and mfs by MsrR. Figure S6. Reversible inhibition of the DNA binding activity of MsrR by H2O2 and role of Cysteine residue. Figure S7. Determination of the apparent KD values of MsrR and MsrR:C62S for PmsrR. Figure S8. Mutations in the predicted MsrR binding site derepressed the msrR expression. Figure S9. Oxidative stress-dependent structural changes of relevant MsrR in vivo. [file 12934_2020_1444_MOESM1_ESM.docx]

**Additional Data**

**MsrR is a thiol-based oxidation-sensing regulator of the XRE family that modulates *C. glutamicum* oxidative stress resistance**

Meiru Si ^a#*^, Can Chen ^b#^, Jingyi Zhong ^a^, Xiaona Li ^a^, Yang Liu ^a^, Tao Su ^a^, Ge Yang ^a*^

^a^ College of Life Sciences, Qufu Normal University, Qufu, Shandong 273165, China;

^b^ College of Life Science and Agronomy, Zhoukou Normal University, Zhoukou, Henan 466001, China

Running title: An oxidative stress-sensing XRE-type sensor MsrR of *C. glutamicum*

**^#^** These authors contributed equally to this work.

**^*^** Corresponding authors:

Meiru Si, Ge Yang

E-mail: [smr1016@126.com](mailto:smr1016@126.com); yangge100@126.com

Tel: 86-15666758564; 86-13953760056

**Table S1. Bacterial strains and plasmids used in this study.**

| **Strains or plasmids** | **Relevant genotype description** | **References** |
| --- | --- | --- |
| **Strains** | | |
| ***Corynebacterium glutamicum*** | | |
| RES167 | Restriction-deficient mutant of ATCC13032, Δ(*cglIM-cglIR-cglIIR*) | [1] |
| Δ*msrR* | *msrR* deleted in RES167 | This study |
| Δ*3-mst* | *3-mst* deleted in RES167 | This study |
| Δ*mfs* | *mfs* deleted in RES167 | This study |
| ***E. coli*** | | |
| BL21(DE3) | *E. coli* expression host, *hsdS gal* (*λc*I*ts*857 *ind-l* *Sam7 nin-*5 *lac UV5-*T7 gene 1) | Novagen |
| JM109 | *recA1 supE44 endA1 hsdR17 gyrA96 relA1 thi* Δ(*lac-proAB*)F′(*traD36 proABlacI*^q^ *lacΔZM15*) | Stratagene |
| **Plasmids** | | |
| pK18*mobsacB* | Suicide plasmid carrying *sacB* for selecting double crossover in *C. glutamicum*, Km^r^ | [2] |
| pK18*mobsacB-*Δ*msrR* | Construct used for in-frame deletion of *msrR* | This study |
| pK18*mobsacB-*Δ3-*mst* | Construct used for in-frame deletion of *3-mst* | This study |
| pK18*mobsacB-*Δ*mfs* | Construct used for in-frame deletion of *mfs* | This study |
| pK18*mobsacB-P_msrR_::lacZY* | *P_msrR_::lacZY* fusion in pK18*mobsacB* | This study |
| pK18*mobsacB-P_mfs_::lacZY* | *P_mfs_::lacZY* fusion in pK18*mobsacB* | This study |
| pXMJ19 | Shuttle vector (*P_tac_ lacI^q^ pBL1 oriV_C. glutamicum_* pK18 *oriV_E. coli_*) | [3] |
| pXMJ19-His_6_ |  | [4] |
| pXMJ19-*msrR* | *msrR* cloned into pXMJ19 for complementation | This study |
| pXMJ19-3-*mst* | *3-mst* cloned into pXMJ19 for complementation | This study |
| pXMJ19-*mfs* | *mfs* cloned into pXMJ19 for complementation | This study |
| pXMJ19-His_6_-*msrR* | *msrR* cloned into pXMJ19-His_6_ for complementation | This study |
| pXMJ19-*msrR:C62S* | *msrR:C62S* cloned into pXMJ19 for complementation | This study |
| pET28a | Expression vector with N-terminal hexahistidine affinity tag | Novagen |
| pET28a*-msrR* | *msrR* in pET28a | This study |
| pET28a-*msrR:C62S* | *msrR:C62S* in pET28a | This study |

**Additional References**

1. Tauch A, Kirchner O, Löffler B, Götker S, Pühler A, Kalinowski J. Efficient electrotransformation of corynebacterium diphtheriae with a mini-replicon derived from the *Corynebacterium glutamicum* plasmid pGA1. [Curr Microbiol](http://www.ncbi.nlm.nih.gov/pubmed/?term=Efficient+electrotransformation+of+corynebacterium+diphtheriae+with+a+mini-replicon+derived+from+the+Corynebacterium+glutamicum+plasmid+pGA1.). 2002; 45: 362-367.

2. Jakoby M, Ngouoto-Nkili CE, Burkovski A. Construction and application of new *Corynebacterium glutamicum* vectors. Biotechnol Tech.1999;13: 437-441.

3. Karimova G, Pidoux J, Ullmann A, Ladant D. A bacterial two-hybrid system based on a reconstituted signal transduction pathway. [Proc Natl Acad Sci U S A](http://www.ncbi.nlm.nih.gov/pubmed/9576956).1998;95;5752-5756.

4. Liu Y, Chen C, Chaudhry MT, Si M, Zhang L, Wang Y, Shen XH. Enhancing *Corynebacterium glutamicum* robustness by over-expressing a gene, *mshA*, for mycothiol glycosyltransferase. Biotechnol. Lett. 2014; 36:1453–1459.

**Table S2.** Primers used in this study.

| **Primers** | **5’-3’ sequence** |  |
| --- | --- | --- |
| CMsrR-F1 | CGCGGATCCAAAGGAGGACAACCATGACCTCCATTAAAGCAACCC(*Bam*HI) | For cloning *msrR* wild type and mutants into pXMJ19 |
| CMsrR-R1 | CAAGAATTCTTAGGGAGCTATAACCGTTTC(*Eco*RI) |  |
| CMsrR-F2 | CGCGGATCCATGACCTCCATTAAAGCAACCC(*Bam*HI) | For cloning *msrR* wild type and mutants into pXMJ19-His_6_ |
| CMsrR-R2 | ACGCGTCGACTTAGGGAGCTATAACCGTTTC(*Sal*I) |  |
| OMsrR-F | CAAGAATTCGGTGGTGGTGGTGCTCGAGTTAGCGCCAACCGCTCGT (*Eco*RI) | For cloning *msrR* wild type and mutants into pET28a |
| OMsrR-R | CCGCTCGAGTGGGTCGCGGATCCGAATTCGTGAAGATCGATGAATTGATTGCTC (*Xho*I) |  |
| DMsrR-F1 | CAAGAATTCCTATGACATGATTACGAATTCGTGATGATTTCCGGTTCGTCGAC (*Eco*RI) | To generate pK18*mobsacB-*Δ*msrR* |
| DMsrR-R1 | GTGGAGGCTGTCGCTGGTGCCATCGCCGACCCGC |  |
| DMsrR-F2 | GATGGCACCAGCGACAGCCTCCACATGTTCGAGG |  |
| DMsrR*-*R2 | CGCGGATCCCAGGTCGACTCTAGAGGATCCGCGGATTTCTTCTGGTGATTTG (*Bam*HI) |  |
| D3-MST-F1 | CGCGGATCGCGGGTCGGCGATGGCACCAGCGAG | To generate pK18*mobsacB-*Δ*3-mst* |
| D3-MST-R1 | CAGAAAGCAGCGAGAACAGTTTG |  |
| D3-MST-F2 | CAAACTGTTCTCGCTGCTTTCTGGCACGCGGGGCTAACCGGTGCGAGC |  |
| D3-MST*-*R2 | CCCAAGCTTTCACGGGCACGCCCGCCAAATCCAC (*Hin*dIII) |  |
| DMFS-F1 | CGCGGATCCCGGATCGTTGATATTCCACCGCTCAAAAC (*Bam*HI) | To generate pK18*mobsacB-*Δ*mfs* |
| DMFS-R1 | ACCAGGGCACTGAGACAATAAAACACGAG |  |
| DMFS-F2 | CTCGTGTTTTATTGTCTCAGTGCCCTGGTGTGGGTGTTTTGGCTGGAGGAAGACGGCC |  |
| DMFS*-*R2 | CAAGAATTCAGGATCACGCCCCAGATGGTGTCCGCG (*Eco*RI) |  |
| C3-MST-F | CGCGGATCAAAGGAGGACAACCATGACCGTGTTGATTTCTCCGTC (*Bgl*II) | For cloning *3-mst* into pXMJ19 |
| C3-MST-R | CCGGAATTCCAGCCAAGCTGAATTCTTAGGGGCGGGGCCACTTCATATCG (*Eco*RI) |  |
| CMFS-F | CGCGGATCAAAGGAGGACAACCGTGTCCACGTTTCATAAAGTTCTG (*Bam*HI) | For cloning *mfs* into pXMJ19 |
| CMFS-R | CCGGAATTCTTACTTCTCAGCAGCTCCCGCAATG (*Eco*RI) |  |
| OMsrR-C62S-F | CGCACCACGCCT*C*CGATCCCTTTGC | To generate *msrR:C62S* DNA fragment |
| OMsrR-C62S-R | GCAAAGGGATCG*G*AGGCGTGGTGCG |  |
| *P_msrR_*-F | TCCCCCGGGGAACAGAAATCCAGTGGTGACG (*Sma*I) | To generate pK18*mobsacB-P_msrR_::lacZY* and the 232-bp *msrR* promoter |
| *P_msrR_*-R | ACTAGTATCGATCTTCACAACAAACCATG (*Spe*I) |  |
| lacZY-F1 | CATGGTTTGTTGTGAAGATCGATACTAGT ATGACCATGATTACGGATTC(*Spe*I) |  |
| lacZY-R | AAAACTGCAGTTAAGCGACTTCATTCACCTG(*Pst*I) |  |
| *P_mfs_*-F | TCCCCCGGGACCGGAACGCCTGGAGATGCGAG (*Sma*I) | To generate pK18*mobsacB-P_mfs_::lacZY* and the 235-bp *mfs* promoter |
| *P_mfs_*-R | ACTAGTATGAAACGTGGACACGGGGTGTC (*Spe*I) |  |
| lacZY-F2 | GACACCCCGTGTCCACGTTTCATACTAGT ATGACCATGATTACGGATTC(*Spe*I) |  |
| QMsrR-F | GATCGATGAATTGATTGCTCTC | RT-PCR |
| QMsrR-R | CGCTGGTGCCATCGCCGACCCG |  |
| Q3-MST-F | GGCCGCAACCCACTGCCACCGCTG | RT-PCR |
| Q3-MST-R | CCGTCAAGGATGCGAACGTTGGGC |  |
| QMFS-F | GGCTGGAGGAAGACGGCCTGAG | RT-PCR |
| QMFS-R | CGCGTTCAGCTTCAGGAACCAAC |  |
| EMsrR-F | AACAAACCATGCTAATCATCAC | To produce the 162-bp EMSA promoter DNA |
| EMsrR-R | GTGGACACGGGGTGTCTCCTAAAG |  |
| Control-F | GGAGTTCCTGGGACGTTTTCCC | To produce the 162-bp EMSA control DNA |
| Control-R | TAAGTTTGTCCTGCGCCTGCTG |  |
| 16 S rRNA-F | ACCCTTGTCTTATGTTGCCAG | RT-PCR |
| 16 S rRNA-R | TGTACCGACCATTGTAGCATG |  |
| F1-F | ACAGTTTGTTTCTTACCAGCGTGG | co-transcription |
| F1-R | ATTGATCTACACAGCCTCCACATG |  |
| F2-F | CAGGTGATTCAGCCTTTTTAGC | co-transcription |
| F2-R | CAGTTGCTGGCTGGCATGGAGC |  |

Underlined sites indicated restriction enzyme cutting sites added for cloning. Letters in italic denoted the mutation sites in overlap PCR for site-directed mutation.


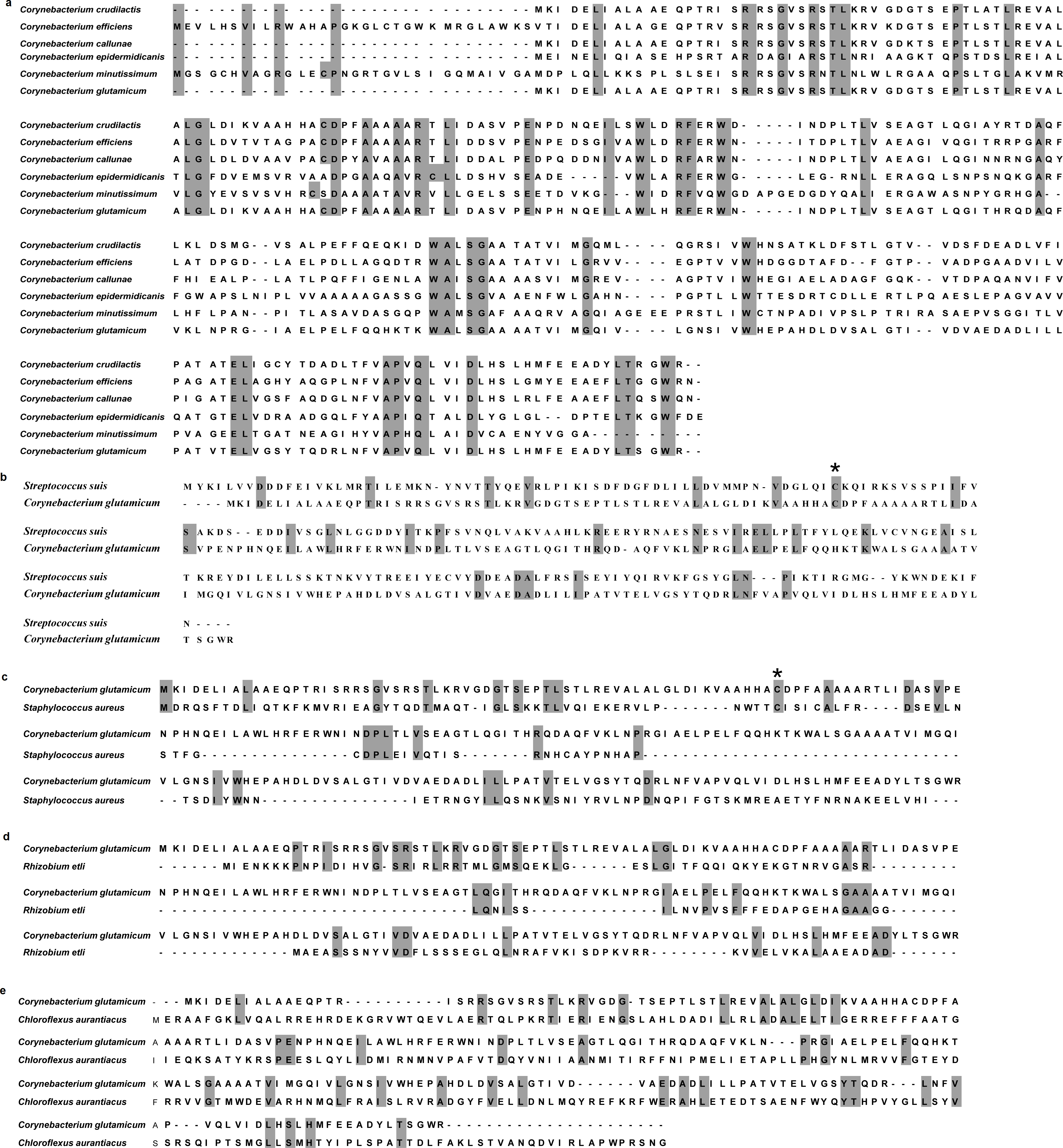

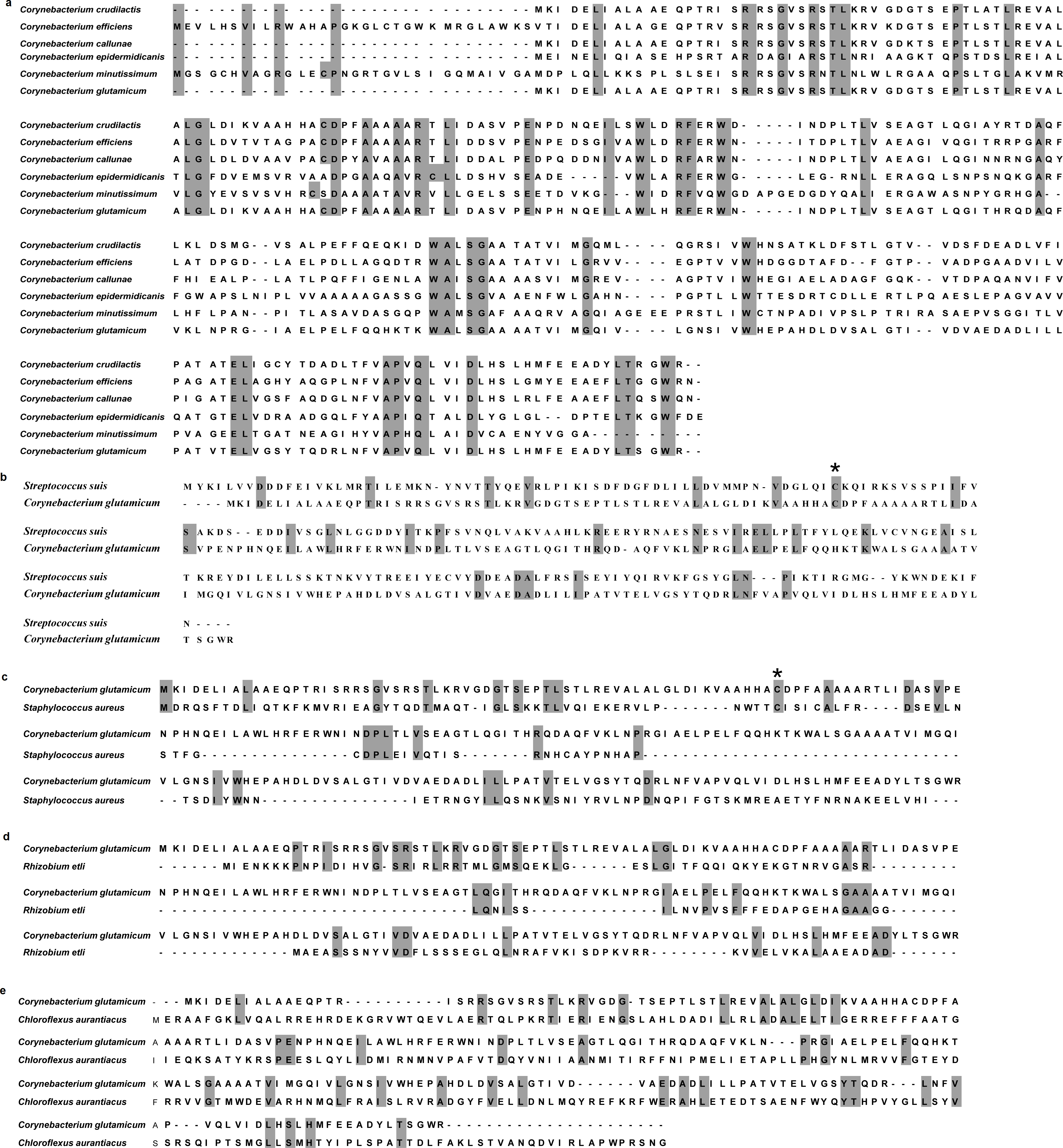


**Figure S1** **Multiple sequence alignment of MsrR with XREs from other organisms. a** Multiple sequence alignment of MsrR with XREs from several species of the genera *Corynebacterium.* Residues that were identical in all or at least 2 of the 5 sequences were depicted on the gray background. Reference sequences were retrieved from the NCBI Database, including *C. glutamicum* ATCC MsrR (NP 601970), *C. crudilactis* XRE (ANE04939); *C. efficiens* XRE (BAC19417); *C. callunae* XRE (AGG67769); *C. epidermidicanis* XRE (AKK03731); *C. minutissimum* XRE (SQH98133). (**b-e**) Multiple sequence alignment of MsrR with XREs from *Streptococcus suis* (**b**), *Staphylococcus aureus* (**c**), *Rhizobium etli* (**d**), and *Chloroflexus aurantiacus* (**e**). Residues that were identical in 2 sequences were depicted on the gray background. Reference sequences were retrieved from the NCBI Database, including *C. glutamicum* ATCC MsrR (NP 601970), *S. suis* SrtR (ANJ64386); *S. aureus* XdrA (BAB42933); *R. etli* RHE-CH00371 (ABC89193); *C. aurantiacus* MltR (YP_001635876). Cys was pointed out by a black star.


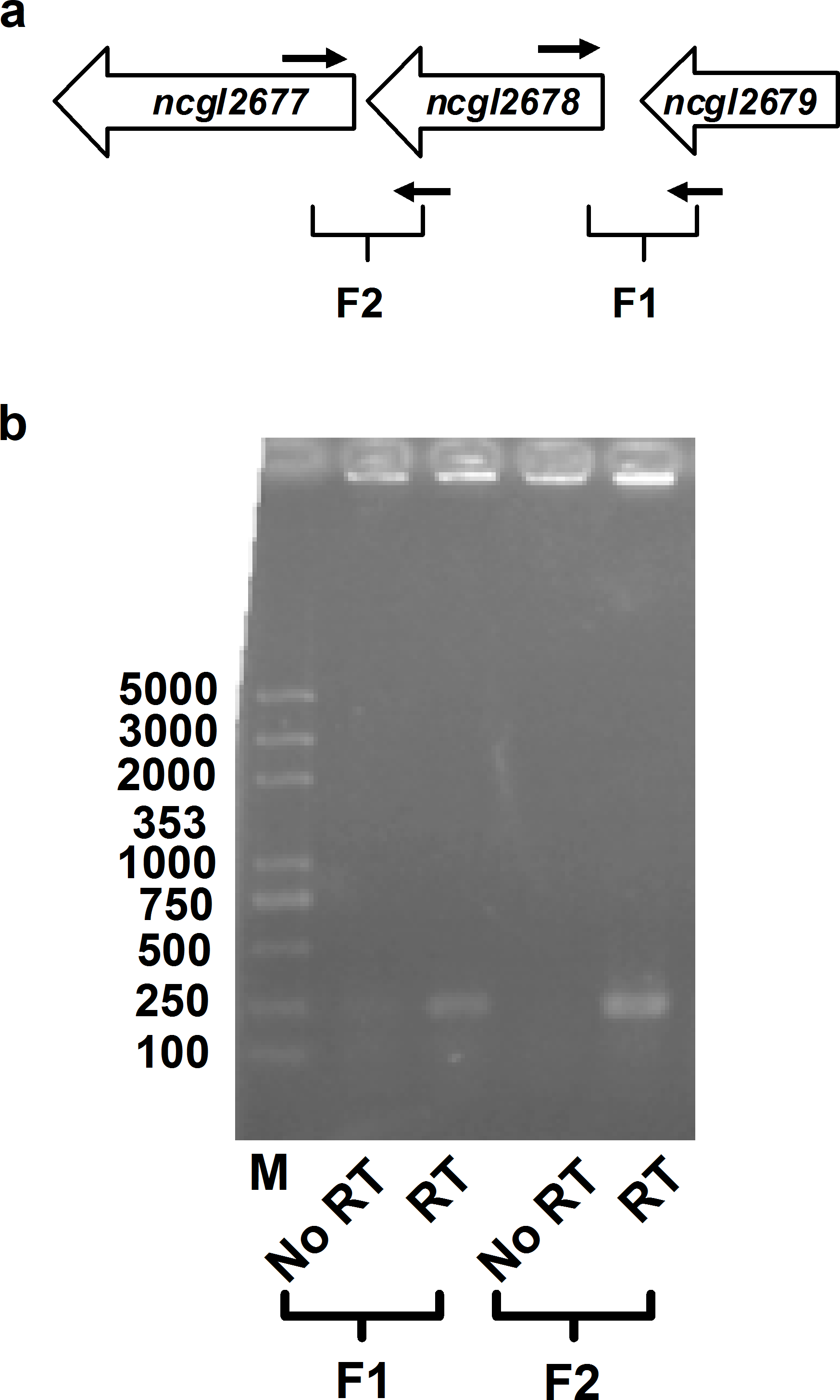


**Figure S2** **Assays for the *ncgl2679*-*ncgl2678*-*ncgl2677* co-transcription by reverse transcription PCR.** A: The operon structure of *ncgl2677*-*ncgl2678-ncgl2679* primer where designed for assays and indicated by blank arrows. B: Reverse transcription PCR assays for *ncgl2677*-*ncgl2678-ncgl2679* co-transcription. Negative control PCR reactions omitted the initial reverse transcription step (No-RT). PCR procedure was as follows: reactions were denatured at 95°C for 50 s, annealed at 58°C for 40 s, extended at 72°C for 30 s, and repeated 30 cycles.


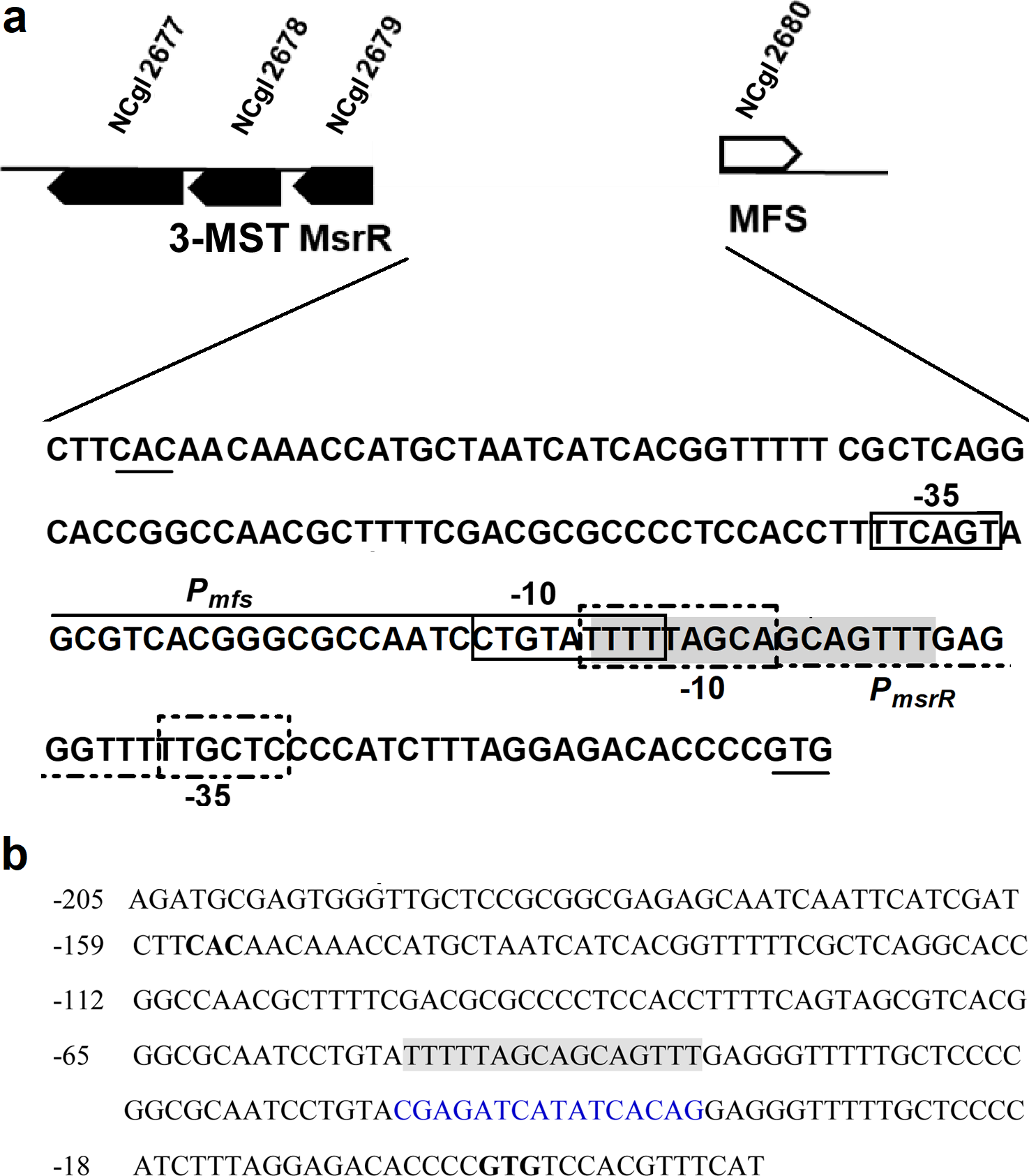


**Figure S3** **Detailed genetic maps of the regulatory region of MsrR.** **a** The intergenic region was downstream of *msrR* gene. The upper lane of the intergenic region was the left-oriented *msrR*-*3-mst* operon, while the lower lane was the right-oriented *mfs* gene. Gray shaded box indicated the putative binding site of MsrR. The deduced -35 and -10 promoter regions found by PROM-Prediction of bacterial promoters were boxed. Dotted box and full box expressed the putative promoter regions of *msrR* and *mfs*. The start codons of the *msrR* and *mfs* were underlined. **b** Prediction of a MsrR binding site in the promoter region of *msrR*. Putative MsrR binding site was indicated with the gray shaded box found by the online software Virtual Footprint. The sequences used to replace the putative binding site were shown in blue below the promoter sequence. The start codons of *msrR* and *mfs* were marked in bold letters.


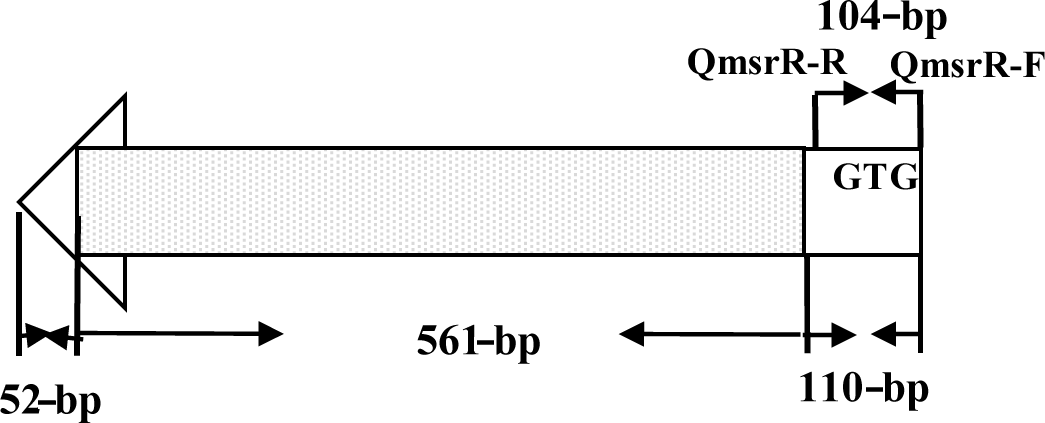


**Figure S4** **104-bp *msrR* transcript (from the translational start codon (GTG) of *msrR* gene to 104th nucleotide) was amplified from the remaining 110-bp *msrR* ORF (open reading frame) in Δ*msrR* mutant with primers QmsrR-F and QmsrR-R.** *msrR* ORF was marked by open arrows and the deleted region was in gray.


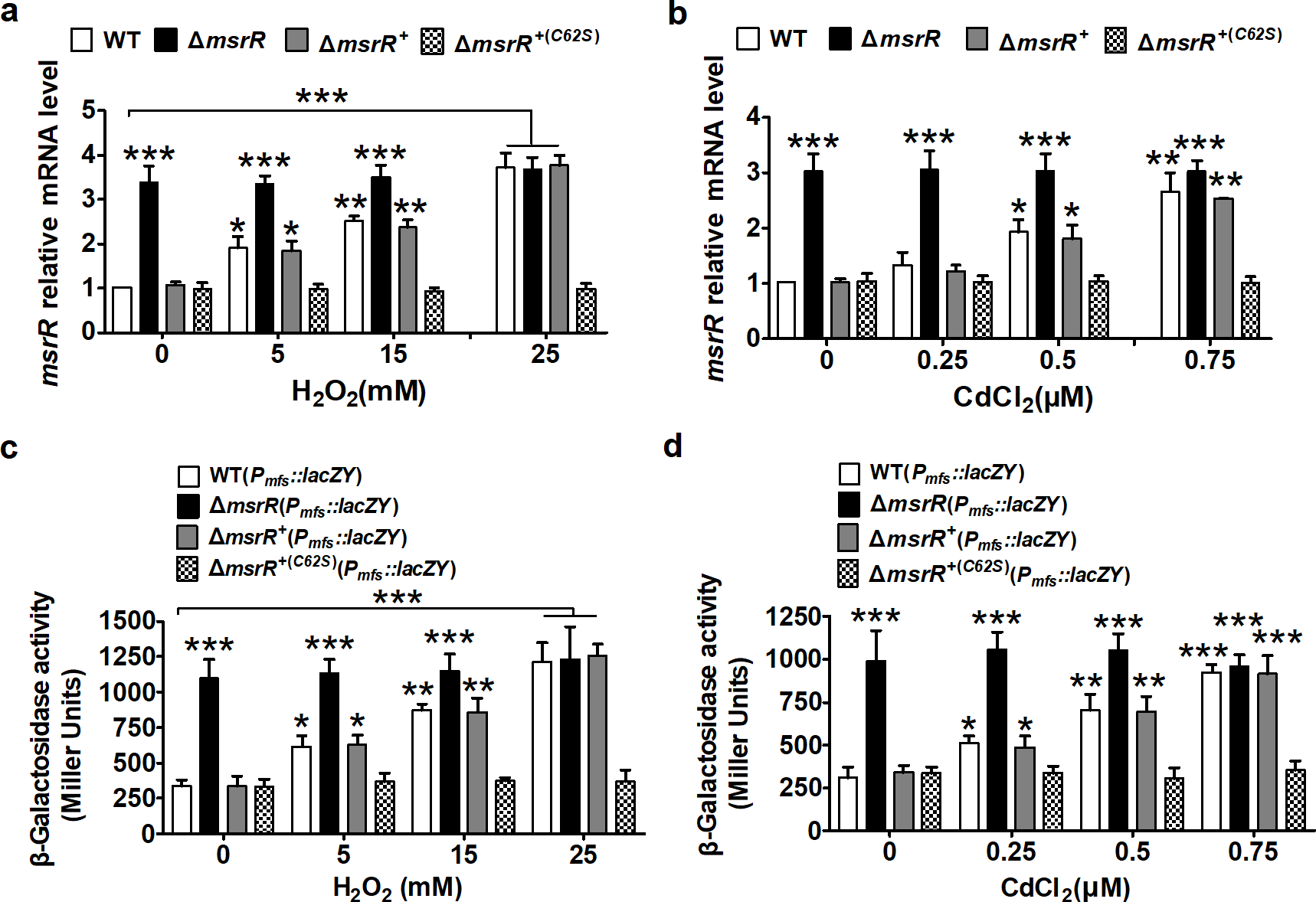


**Figure S5** **Negative regulation of *msrR* and *mfs* by MsrR**. **a and b** Quantitative RT-PCR analyses of *msrR* expression in WT, Δ*msrR* mutant, and the complementary strain Δ*msrR*^+^ under indicated conditions. Data show the averages of three independent experiments; the standard deviation was indicated by bars. The mRNA levels were presented relative to the value obtained from WT cells without stress treatment. Relative transcript levels of WT strains without stress treatment were set at a value of 1.0. *: P≤0.05; ***P* < 0.01; ****P* < 0.001. **c and d** β-galactosidase analyses of *mfs* promoter (*P_mfs_*) activity by using the transcriptional *P_mfs_::lacZY* chromosomal fusion reporter expressed in WT, Δ*msrR* mutant, and the complementary strain Δ*msrR*^+^. Data show the averages of three independent experiments; the standard deviation was indicated by bars. *: P≤0.05; ***P* < 0.01; ****P* < 0.001.


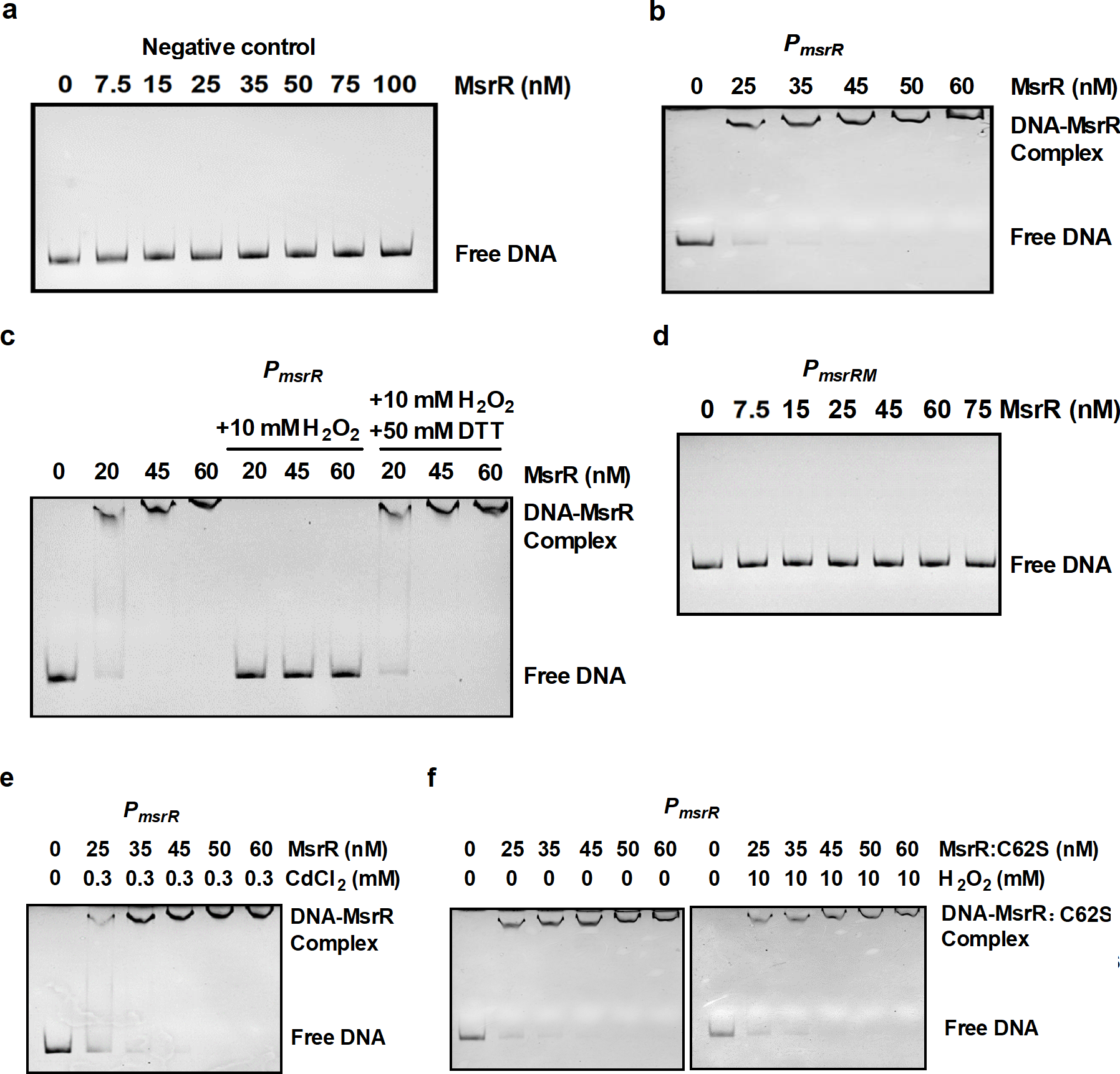

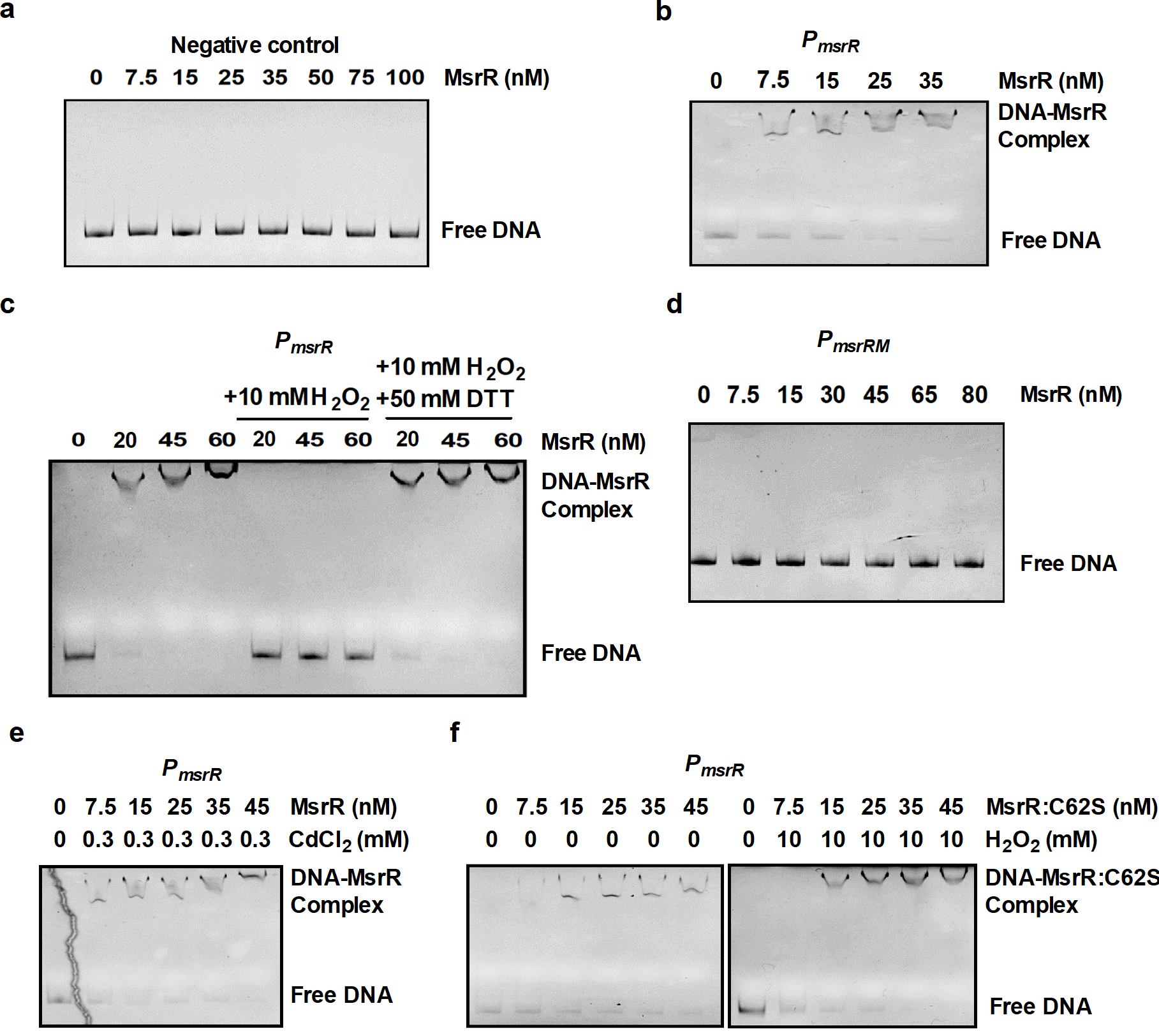


Repeat experiment 1 Repeat experiment 2

**Fig. S6 Reversible inhibition of the DNA binding activity of MsrR by H_2_O_2_ and role of Cysteine residue. a** The interaction between His_6_-MsrR and DNA fragments amplified from MsrR’s ORF. **b** The interaction between His_6_-MsrR and the promoter fragment in the intergenic region between *msrR* and *mfs* (named *P_msrR_*). **c** Inhibition of the DNA binding activity of MsrR by H_2_O_2_ and reversal of the inhibition by DTT. MsrR was prepared in three different concentrations, and aliquots were taken for EMSAs (control). Then H_2_O_2_ was added to each MsrR sample to a final concentration of 10 mM, and again aliquots were taken for EMSAs. In the next step DTT was added to a final concentration of 50 mM, and aliquots were taken for EMSAs. All aliquots were incubated in binding buffer, pH 8.0, with 40 ng *P_msrR_* and then separated on an 8% native polyacrylamide gel. **d** The interaction between His_6_-MarR and the promoter mutating the predicted MsrR binding region (*P_msrRM_*). **e** CdCl_2_ was added to the binding reaction mixture to a final concentration of 0.3 mM, and the interaction between His_6_-MsrR and the *msrR* promoter (*P_msrR_*) were performed. **f** The interaction between the mutated derivatives MsrR:C62S and *P_msrR_* in the presence or absence of 10 mM H_2_O_2_. Data show two replicates done in triplicate.


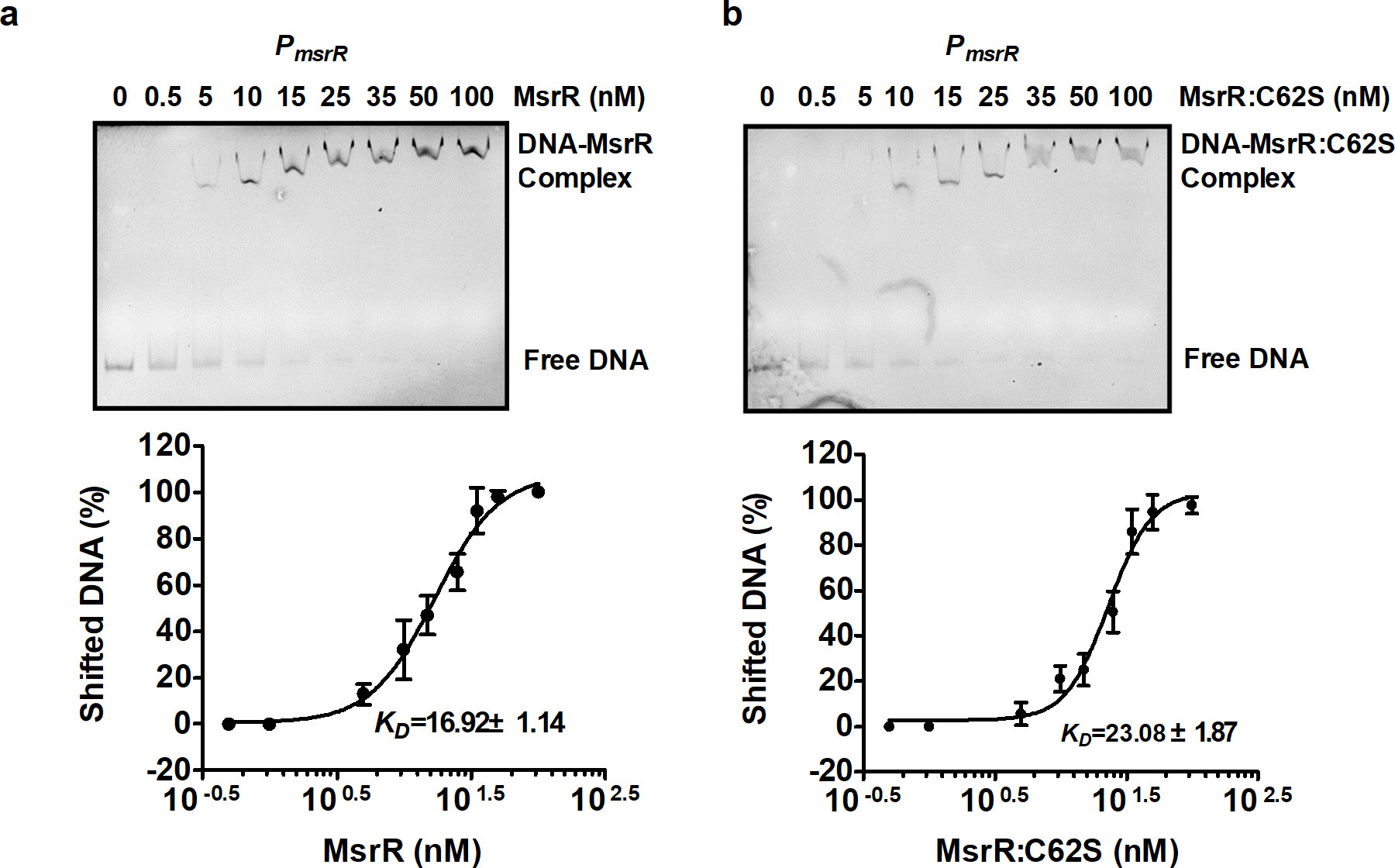


**Figure S7** Determination of the apparent *K_D_* values of MsrR and MsrR:C62S for *P_msrR_*. *P_msrR_* was incubated with increasing MsrR (a) or MsrR:C62S (b) concentrations, resolved on a 8% native polyacrylamide gel, and stained with GelRed^TM^. At least three independent gels were performed for each binding site. The bands were quantified using ImageQuant software (GE Healthcare), and the percentage of shifted DNA was calculated from three independent gels. These values were plotted against the MsrR concentration in log_10_ scale, and a sigmoidal fit was performed. The turning point of the curve was defined as the apparent *K_D_* value.


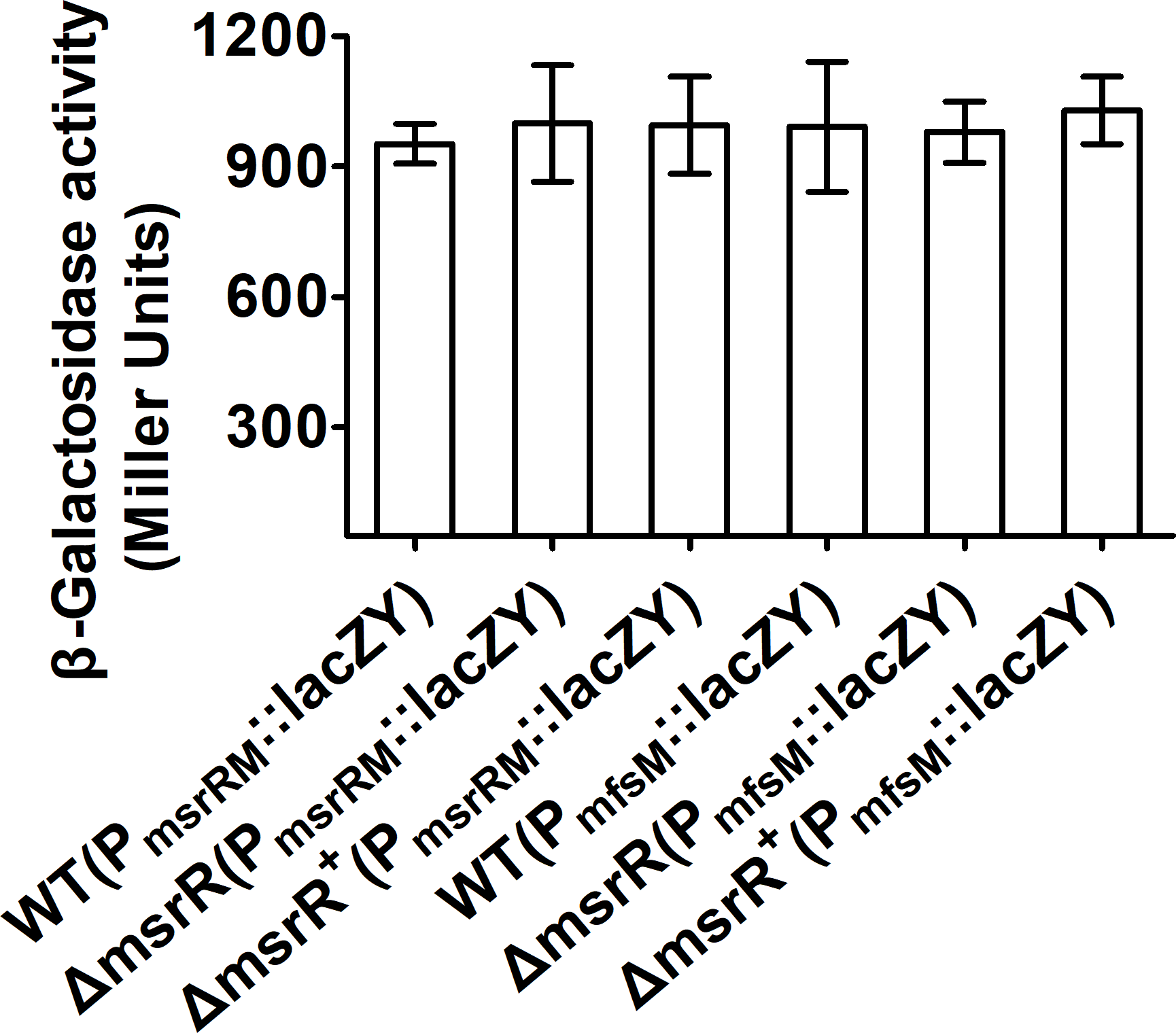


**Figure S8** **Mutations in the predicted MsrR binding site derepressed the *msrR* expression.** β-galactosidase activity was measured in indicated bacterial strains. Relative levels of transcripts were presented as the mean values ± SD calculated from three sets of independent experiments.


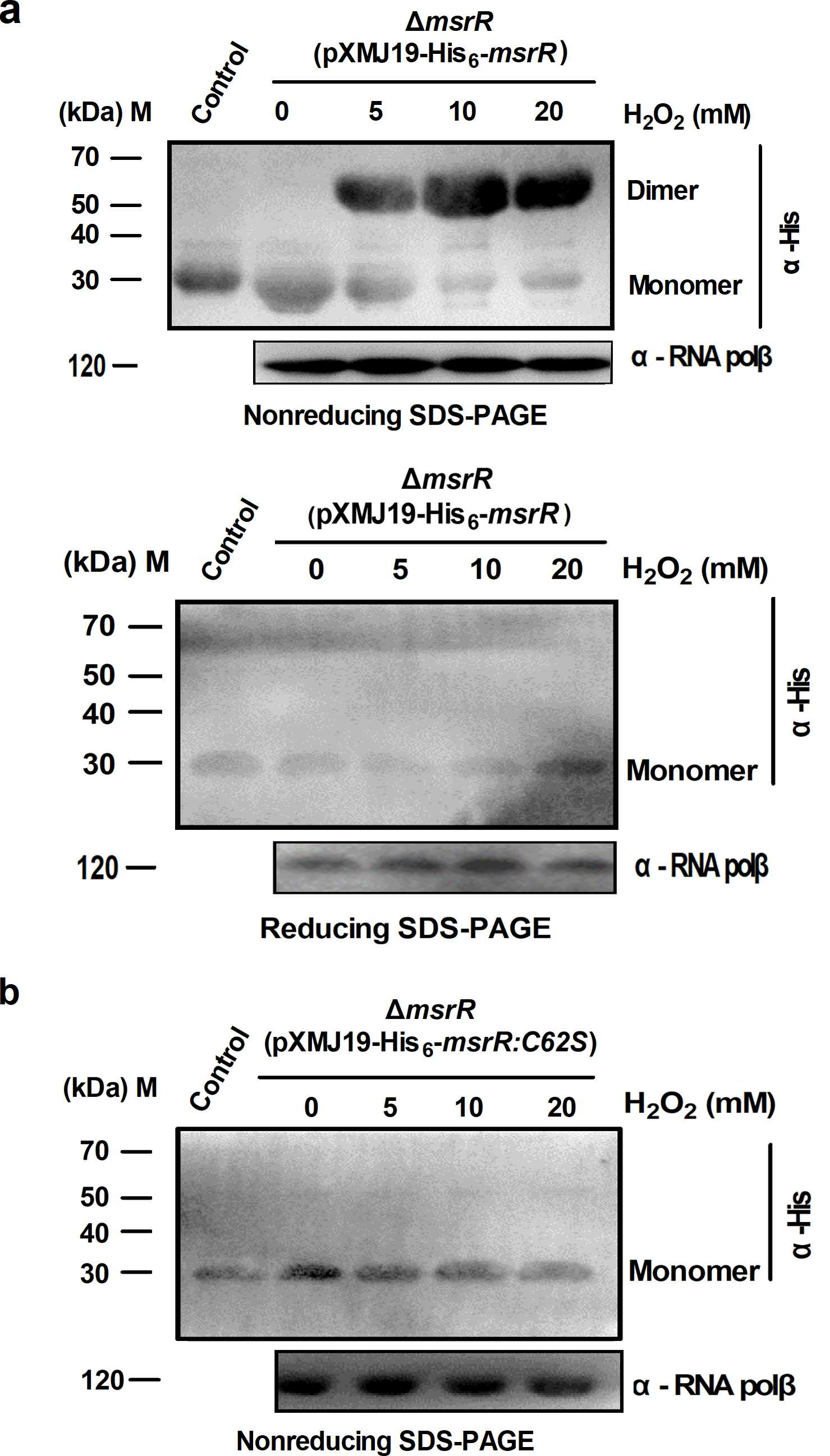

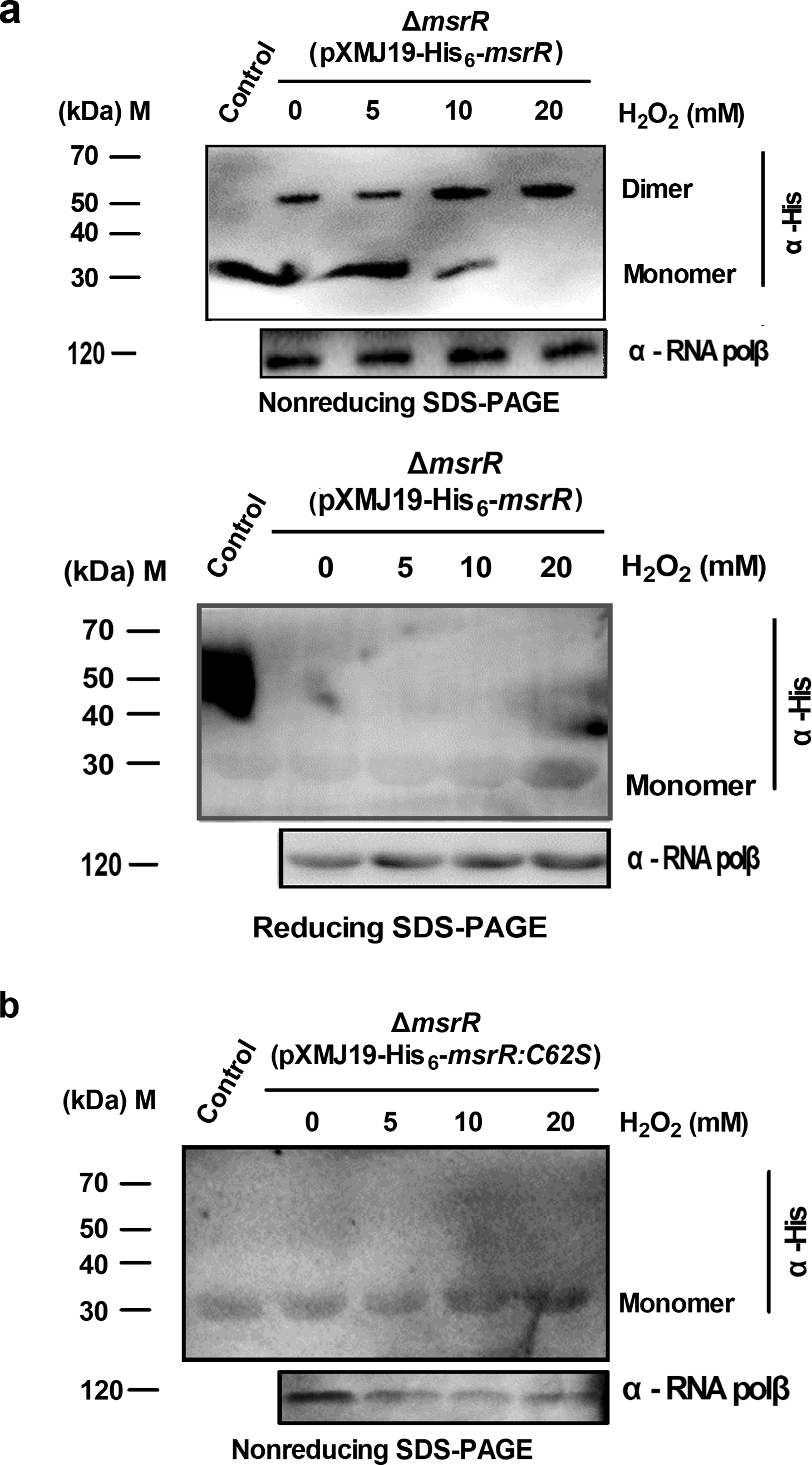


Repeat experiment 1 Repeat experiment 2

**Figure S9 Oxidative stress-dependent structural changes of relevant MsrR *in vivo*.** MsrR (**a**) and MsrR:C62S (**b**) extracted from cells exposed to different concentrations of H_2_O_2_ for 30 min were resolved on nonreducing or reducing SDS-PAGE, and analyzed with Western blotting by using the anti-His antibody. RNA polymerase β (RNA polβ) was used as a loading control. Data show two replicates done in triplicate.
